# Baseline state for pulmonary vasculature with pulmonary arterial hypertension: effect of geometric remodeling and metabolic shift

**DOI:** 10.1007/s10237-026-02076-4

**Published:** 2026-06-10

**Authors:** Haritha N. Mullagura, Hamidreza Gharahi, C. Alberto Figueroa, Seungik Baek

**Affiliations:** 1 Department of Mechanical Engineering, Michigan State University, East Lansing, MI USA; 2 Section of Vascular Surgery, Department of Surgery, University of Michigan, Ann Arbor, MI USA; 3 Department of Biomedical Engineering, University of Michigan, Ann Arbor, MI USA

**Keywords:** Hemodynamics, Mechanical homeostasis, Metabolic shift, Pulmonary hypertension

## Abstract

Pulmonary arterial hypertension (PAH) is a complex disease characterized by chronically elevated pulmonary arterial pressure, with early onset and progression linked to structural, metabolic, and morphological changes in the pulmonary vasculature. Understanding the interplay between hemodynamics and arterial wall mechanics is essential to capture the pathology of the distal vasculature in PAH. This study aims to develop a data-driven framework that establishes a baseline state of PAH vasculature, incorporating key features of arterial wall constituents, geometry, and their interaction with PAH-specific hemodynamics. Illustrative examples of symmetrically bifurcating arterial trees are used to define representative baseline characteristics of PAH-affected pulmonary arteries. Compared with healthy homeostatic vasculature, the computational results demonstrate pronounced geometric and mechanical alterations: Arterial stiffness increases from approximately 7–10 kPa in healthy arteries to 300–800 kPa in PAH, representing a ~ 40–85 times increase across generations. Because wall thickening is imposed from histological measurements while outer diameter is preserved, the diameter-to-thickness ratio (D/h) decreases from ~ 14 in healthy arteries to ~ 3.8 in PAH, reflecting severe lumen narrowing and medial hypertrophy. In addition, the metabolic energy cost per unit length in PAH is more than double that of healthy arteries when assuming unchanged metabolic consumption per unit volume, whereas enforcing equal total energy cost yields a reduced per-volume metabolic consumption of ~ 450–500 W/m^3^. These findings suggest that maintaining constant metabolic consumption per unit volume would impose excessive energetic demand on the pulmonary vasculature in PAH, whereas redistribution of metabolic expenditure through altered wall composition may represent a more physiologically plausible adaptation. Furthermore, this framework provides a quantitative baseline state for PAH vasculature and lays the groundwork for future integration of growth-and-remodeling analyses and pharmacological pathway modeling to evaluate treatment response.

**Supplementary Information:**

The online version contains supplementary material available at 10.1007/s10237-026-02076-4.

## Introduction

Pulmonary arterial hypertension (PAH) is a multifaceted vascular disease marked by progressive vascular remodeling, resulting in narrowing of the pulmonary arteries and increased pulmonary vascular resistance (PVR). These changes cause elevated afterload on the right heart, leading to right heart failure if left untreated (Thenappan et al. 2018b). These pathological changes include endothelial cell (EC) proliferation, smooth muscle cell (SMC) hypertrophy, and excessive collagen deposition in the extracellular matrix (ECM) (Chazova et al. 1995; Thenappan et al. 2018a). These factors contribute to thickened and stiffened arterial walls, fundamentally altering the mechanical behavior of the pulmonary vasculature and pulmonary hemodynamic circulation. Clinical imaging is, however, limited in its ability to detect the vascular features of small vessels and human tissue samples from PAH patients are scarce (Shahin et al. 2022). To bridge this gap, computational modeling of pulmonary arterial vasculature has emerged as a valuable tool, offering insight into the relationship between pulmonary geometrical features and hemodynamic function, and complex vascular remodeling processes, despite the limited available experimental data (Hunter et al. 2010b; Qureshi and Hill 2015; Ebrahimi et al. 2022; Szafron et al. 2023).

More recent efforts have focused on PAH-specific models that incorporate stress-mediated growth and remodeling (G&R), such as those that simulate changes in pulmonary artery geometry, mass fraction, and homeostatic stress during PAH progression (Szafron et al. 2023). For a maladaptive alteration case in the pulmonary vascular tree associated with PAH, an additional variable representing an inflammatory stimulus is introduced and incorporated into the G&R function to simulate the progression of vascular maladaptation in PAH. However, a key limitation of these models is that they do not account for metabolic changes, such as the Warburg effect and mitochondrial dysfunction, which play a critical role in PAH progression (Tuder et al. 2007; Paulin and Michelakis 2014; Maron and Leopold 2015).

Alongside structural remodeling, emerging evidence suggests that metabolic dysfunction plays a pivotal role in the progression of PAH. One of the key drivers of this metabolic shift is the upregulation of hypoxia-inducible factor (HIF), which orchestrates cellular responses to chronic hypoxia by altering energy metabolism (Rai et al. 2008; Pullamsetti et al. 2020). Under hypoxic conditions, cells, including pulmonary artery smooth muscle cells (PASMCs), transition from mitochondrial oxidative phosphorylation to glycolysis, even in the presence of oxygen—known as the Warburg effect (Paulin and Michelakis 2014; Shi et al. 2020). This metabolic shift promotes increased glucose uptake and lactate production, which supports the hyperproliferation of PASMCs and endothelial cells, both of which contribute to vascular remodeling. Mitochondrial dysfunction in PAH further exacerbates this metabolic reprogramming by decreasing the activity of electron transport chain complexes I–III, impairing oxidative phosphorylation and other associated mechanisms, which leads to diminished ATP production (Shi et al. 2020; Riou et al. 2023). This shift to glycolysis is less energy-efficient, producing only 2 molecules of ATP per glucose molecule, compared to 36 ATP molecules generated by oxidative phosphorylation (Park et al. 2014). The metabolic alterations in PAH, therefore, play a critical role in driving both structural and functional changes in the pulmonary arteries, adding complexity to the task of accurately modeling PAH vasculature, as these metabolic shifts directly influence cellular proliferation, apoptosis resistance, and vascular remodeling (Xu et al. 2021).

Therefore, understanding of the relationship between metabolic processes and pulmonary vascular wall remodeling is important for both healthy and diseased states. In fact, Murray’s law establishes a relationship between mechanical stress (e.g., wall shear stress) and metabolic energy consumption in the cardiovascular system (Taber 1998; Lindström et al. 2015). Particularly, in healthy pulmonary vasculature, computational frameworks have been established based on the understanding of the metabolic energy optimization, in which Gharahi et al. (2023) presented a homeostatic optimization framework for a healthy pulmonary vasculature using an extension of Murray’s law that optimizes geometry by incorporating metabolic energy costs and mechanical equilibrium constraints. Specifically, an iterative optimization, which integrates a metabolic cost function minimization, the stress equilibrium, and hemodynamics, has been performed at the slow timescale, while in the fast timescale, the pulsatile blood flow dimensional dynamics are described by a Womersley’s deformable wall analytical solution. The results of the computational framework were compared with diverse literature data on morphometry, structure, mechanical behavior of the pulmonary artery, and blood flow through the 1D vasculature. Nonetheless, the previous study was predicated on the assumption that the metabolic energy consumption of SMC and collagen fiber is unaltered on the vessel for a healthy subject. For PAH, the metabolic energy optimization with fixed metabolic energy consumption of SMC and collagen fiber is not an appealing choice, given the significance of metabolic alteration in PAH. However, quantitative data on metabolic energy consumption in vivo of the arterial vasculature are lacking, presenting a significant restriction on the use of the same optimization approach.

To address these limitations, we propose to develop a data-driven baseline model of PAH vasculature that integrates available geometric, pathological, and hemodynamic data from PAH patients. Baseline, in this context, refers to a state where the arterial geometry, hemodynamics, and wall properties represent those of a typical PAH patient. This baseline model will serve as a reference point from which changes induced by different PAH treatments can be measured. By establishing this baseline state, we aim to capture the specific characteristics of PAH-affected vasculature, allowing for the evaluation of treatment efficacy through the comparison of arterial and hemodynamic responses to interventions. This framework provides a critical foundation for assessing how different therapies modify vascular structure and function relative to this initial, disease-representative state.

The specific objectives of this work are twofold. Using the existing literature of experimental morphometric, pathological, and hemodynamic data, we first construct a symmetrically bifurcating arterial tree, based on two assumptions: constant and varying wall constituents. This baseline model will allow us to simulate the mechanical behavior of PAH-affected arteries and study the impact of different hemodynamic conditions on vessel stiffness and stress. Second, although the metabolic costs of maintaining a healthy arterial wall have been well characterized, there remains a notable gap in our knowledge regarding these costs in PAH. To address this, we assess the metabolic expenditure required to sustain the arterial vasculature in PAH by evaluating two conceptual conjectures regarding metabolic energy cost: fixed metabolic energy consumption per volume versus variable metabolic energy consumption per volume to sustain total metabolic expenditure. These parallel conjectures enable a direct comparison of their implications and facilitate discussion of the mechanisms underlying metabolic adaptation in PAH.

## Methods

**Fig. 1:**
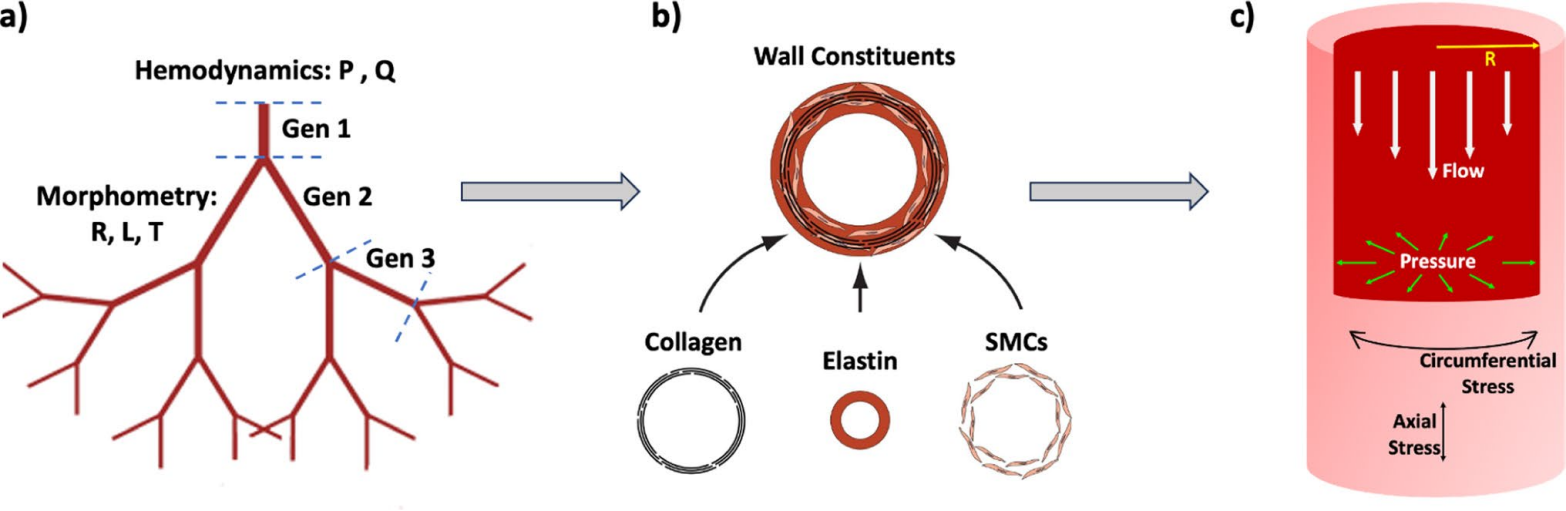
Schematic representation of the establishment of a baseline state for pulmonary arteries with PAH, **a** morphometry (radius, length, and thickness) and arterial tree hemodynamics (pressure and flow) are constructed, **b** metabolic costs of wall constituents (elastin, collagen, and smooth muscle cells) are estimated based on the vessel wall’s mass fraction, and **c** arterial wall material and hemodynamic variables are calculated using a 1D deformable hemodynamics model for the established PAH state

Constructing a 1D pulmonary vasculature and hemodynamic model for PAH is a multi-stage process aimed at capturing the complexity of arterial geometry, hemodynamics, and associated metabolic changes. In this study, we build on the 1D pulmonary vascular model developed by Gharahi et al. (2023), which considered two cases for mass fraction assumptions: (1) a symmetric fractal tree with constant mass fractions of its wall constituents throughout the entire arterial network and (2) a symmetric fractal tree with varying mass fractions across vessels of differing diameters. For the PAH model, we assume that the morphometry of the arterial vasculature tree structure remains unchanged, except for modifications to vessel thickness, internal diameter, and constituent mass fractions, with the values estimated from the literature. Once the PAH model is constructed, key arterial properties of vessels (e.g., stiffness, stress) and hemodynamic variables (e.g., blood flow, pressure, wall shear stress) are estimated throughout the vasculature, as shown in Fig. 1. In addition, metabolic energy costs are calculated under two competing conditions: (1) unaltered metabolic energy cost of collagen and SMCs per unit volume and (2) the same total metabolic cost of the whole pulmonary vasculature as in the healthy vasculature.

### Construction of pulmonary vasculature model for PAH

#### Morphometry of arterial tree

**Fig. 2:**
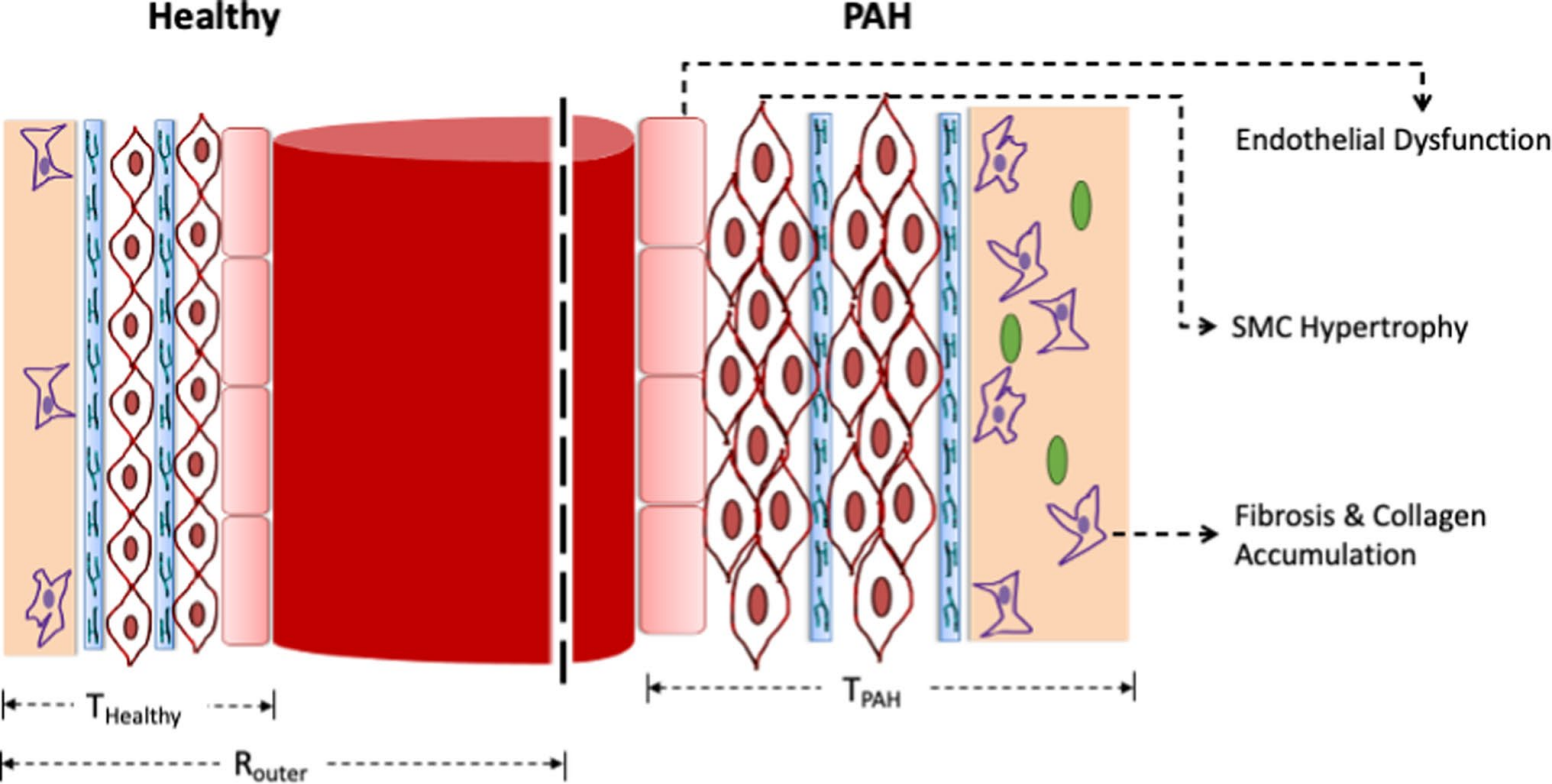
Schematic representation of the longitudinal cross section of a healthy (left) and a PAH (right) arterial wall

To establish the morphometry of the pulmonary arterial tree, we begin with healthy vasculature geometry and then redefine wall thickness based on the reported ratio of wall thickness to external diameter in the PAH literature data of PAH. We assume that the external diameter and length remain consistent between healthy and PAH vasculature, where increased wall thickness observed in PAH results in a reduced inner diameter and lumen. Figure 2 illustrates the morphometric changes in the arterial wall from healthy and PAH-affected pulmonary arteries. In the healthy state (left), the arterial wall consists of thin intimal and medial layers, with a normal lumen size and regular organization of smooth muscle cells and ECM components. Conversely, the PAH state (right) exhibits significant remodeling, characterized by a thickened intimal layer due to endothelial cell proliferation and matrix deposition, as well as medial hypertrophy driven by smooth muscle cell proliferation and hyperplasia. The external diameter, $2*{R}_{outer}$, remains largely unchanged, while the increased wall thickness, ${T}_{PAH}$, reduces the lumen diameter, severely impairing blood flow. This structural remodeling underpins the elevated pulmonary vascular resistance and progressive functional decline observed in PAH.

A brief description of the pulmonary vasculature model is provided. The exploration of vascular network architecture has long been inspired by Murray’s law, which suggests that vessels optimize their diameters to minimize energy dissipation. In our previous work (Gharahi et al. 2023), we extended Murray’s law to compute homeostatic radii for distal pulmonary vasculature, incorporating not only energy optimization for blood flow maintenance and viscosity dissipation energy losses but also the energy for arterial wall maintenance, optimizing both radii and hemodynamics for healthy subjects.

In contrast to the Strahler ordering system (Jiang et al. 1994; Huang et al. 1996; Tamaddon et al. 2016), the morphometry of the tree is described by the generation number in this work, where the third bifurcated vessel from the main pulmonary artery is considered generation 1, and each bifurcation thereafter increments the generation number by 1, resulting in a total of 19 generations, as shown in Fig. 1a. This initial stage focuses on accurately representing the geometric features of pulmonary arteries affected by PAH. The arterial geometry is therefore intended to replicate pathological changes documented in the literature, providing a foundation for subsequent modeling stages (Santos et al. 2002).

Although the real pulmonary arterial tree exhibits asymmetric bifurcation and non-uniform branching, we assume a symmetric structure in this baseline framework to enable direct comparison between healthy and PAH states under controlled geometric conditions. This simplification isolates the influence of wall remodeling and metabolic energy expenditure from topological variability. The metabolic energy cost formulations are algebraic and can be extended to asymmetric trees in future work.

Building on previous work (Gharahi et al. 2023), we maintained the outer diameter scaling similar to that of healthy arteries across different vessel generations. This choice is based on the complexity of reliably extracting generation-specific diameter measurements from human histological data. While pulmonary arteries in vivo exhibit asymmetric bifurcation and non-uniform tapering, we assume a symmetric branching structure and preserve the geometric tapering and connectivity laws from the healthy model. This approach provides a logical and simplified starting point for modeling.

In the current study, we extended the healthy model to a PAH baseline by altering only the vessel wall properties—such as wall thickness, constituent mass fractions (elastin, collagen, SMC), and mechanical stiffness—while keeping the geometric morphometry and branching architecture unchanged. A summary of parameters preserved from the healthy model and those modified in the PAH baseline is provided in **Supplementary Materials (**Table S1**)**. The wall thickness and inner diameter for PAH arteries are then estimated using an empirical relation fitted to histological measurements of PAH vasculature at autopsy (Rol et al. 2017), which provided detailed measurements of the inner and outer diameters of pulmonary arteries in six PAH patients. By fitting the dataset with a linear regression, an empirical relation between the inner and outer diameters is derived, reflecting the average inner-to-outer diameter ratio across these six patients. The individual linear fits for each patient showed variability, with slopes ranging from 0.33 to 0.84, highlighting the heterogeneity of vascular narrowing in PAH. Averaging these slopes led to the inner–outer diameter relation ${D}_{i}= 0.66*{D}_{o}$​, which allows for the estimation of the inner diameter (${D}_{i}$) and wall thickness (H) in PAH vasculature:

1 $${D}_{i}= 0.66*{D}_{o}$$

2 $$H = \frac{{D_{o} - D_{i} }}{2}.$$

This approach enables a more realistic representation of PAH progression by maintaining structural consistency with healthy vasculature while incorporating the specific changes in wall thickness and inner diameter observed in PAH, as informed by the histological data.

The length of the arterial vessel is related to its radius using the relation given in (Olufsen et al. 2012) which is an empirical relation as shown below, using the morphometric data from (Huang et al. 1996), where the length of the arterial vessel (*L*) and the vessel inner radius (*R*) are measured in *mm.*

3 $$L\left( R \right) = 12.4*\left( {R + H} \right)^{1.1} ,$$

where *H* is the vessel wall thickness. The data used in this reference primarily focus on morphometric characteristics in healthy subjects, but the relation has been extended for use in diseased conditions like PAH. This length–radius relationship allows us to model pulmonary artery geometry in both healthy and diseased states, providing insights into how vascular remodeling in PAH affects the overall geometry of the arterial network.

**Fig. 3:**
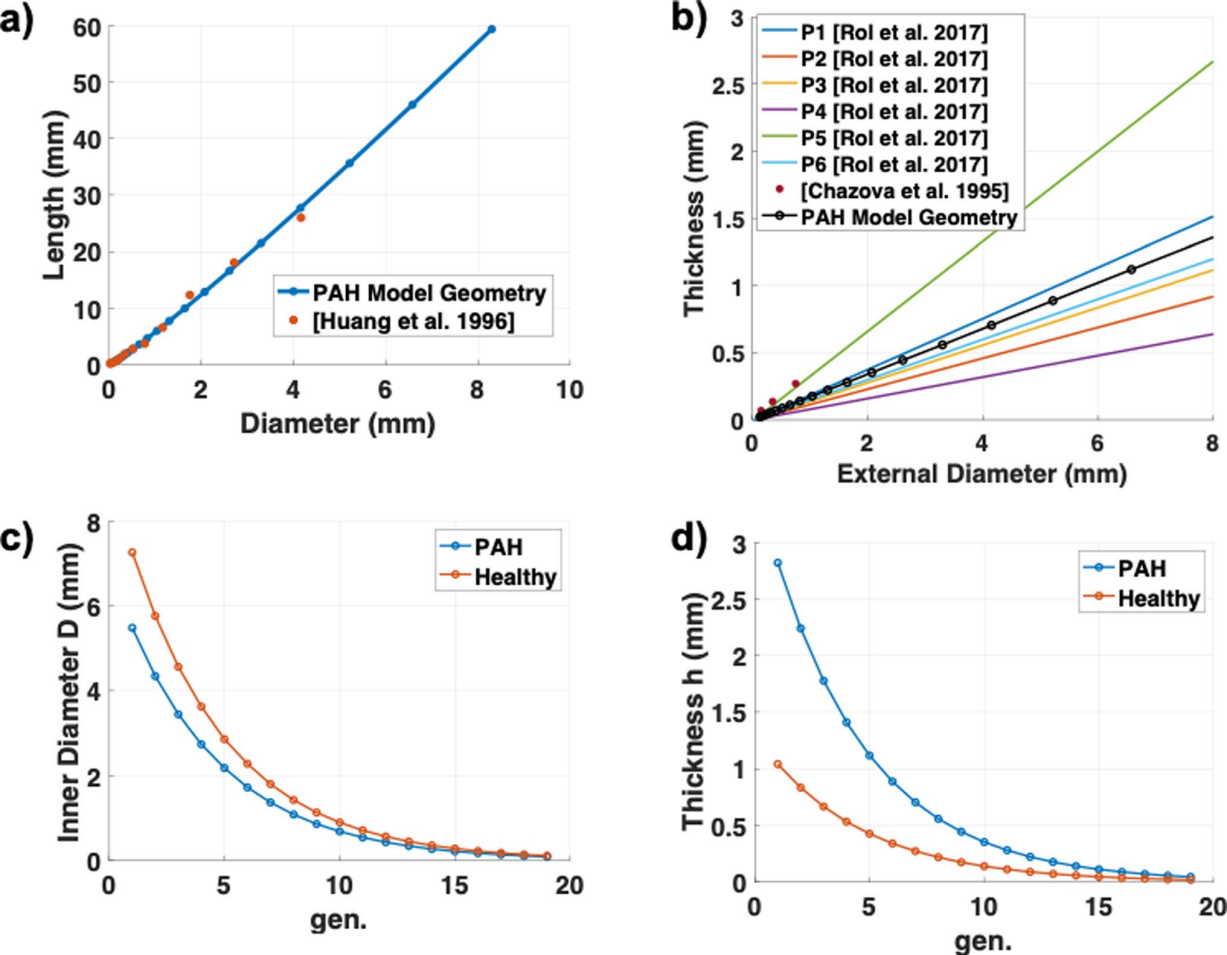
Morphometry of the PAH arterial tree compared to the literature and previously established healthy vasculature. **a** Length of the arterial tree for different diameters as compared with the histological data from (Huang et al. 1996), **b** the thickness of the vessel wall at different external diameters fitted in the model as compared with the PAH data from different patients (Chazova et al. 1995; Rol et al. 2017), **c** inner diameter, and **d** thickness of the arterial tree across different generations are plotted and compared to homeostatic healthy vasculature as described in (Gharahi et al. 2023)

Using the above relations, the morphometry of the entire arterial tree (radius, thickness, and length) is defined for the PAH vasculature, with radius, thickness, and length plotted against external diameter and arterial generation number, shown in Fig. 3.

#### Mass fractions of arterial wall

The two cases of baseline states in this current study are: 1) a symmetric arterial tree with a constant mass fraction, 2) a symmetric arterial tree with varying mass fractions through the vasculature. The total area density of solid constituents (mass per unit area) is specified for each generation of the arterial tree in the current configuration, related to the associated thickness below (Gharahi et al. 2023)

4 $$M_{total}^{n} = h^{n} \left( {1 - \phi_{f} } \right)\rho_{solid} ,$$

where ${h}^{n}$ is the total thickness of *n*th generation arterial wall, ${\phi}_{f}$ is the volume fraction of the interstitial fluid, usually taken as 0.7, and ${\rho}_{solid}$ is the density of the arterial wall, which is considered as 1060 kg/m^3^.

In our model, the vessel wall is assumed to consist of only three constituents—elastin, smooth muscle cells, and collagen fibers, where the total mass per unit area is divided among

5 $$M_{total} = M_{e} + M_{smc} + M_{col} ,$$

where ${M}_{e}$, ${M}_{smc}$, and ${M}_{col}$ represent the mass per unit area cross section of elastin, smooth muscle cells, and collagen fibers, respectively.

**Fig. 4:**
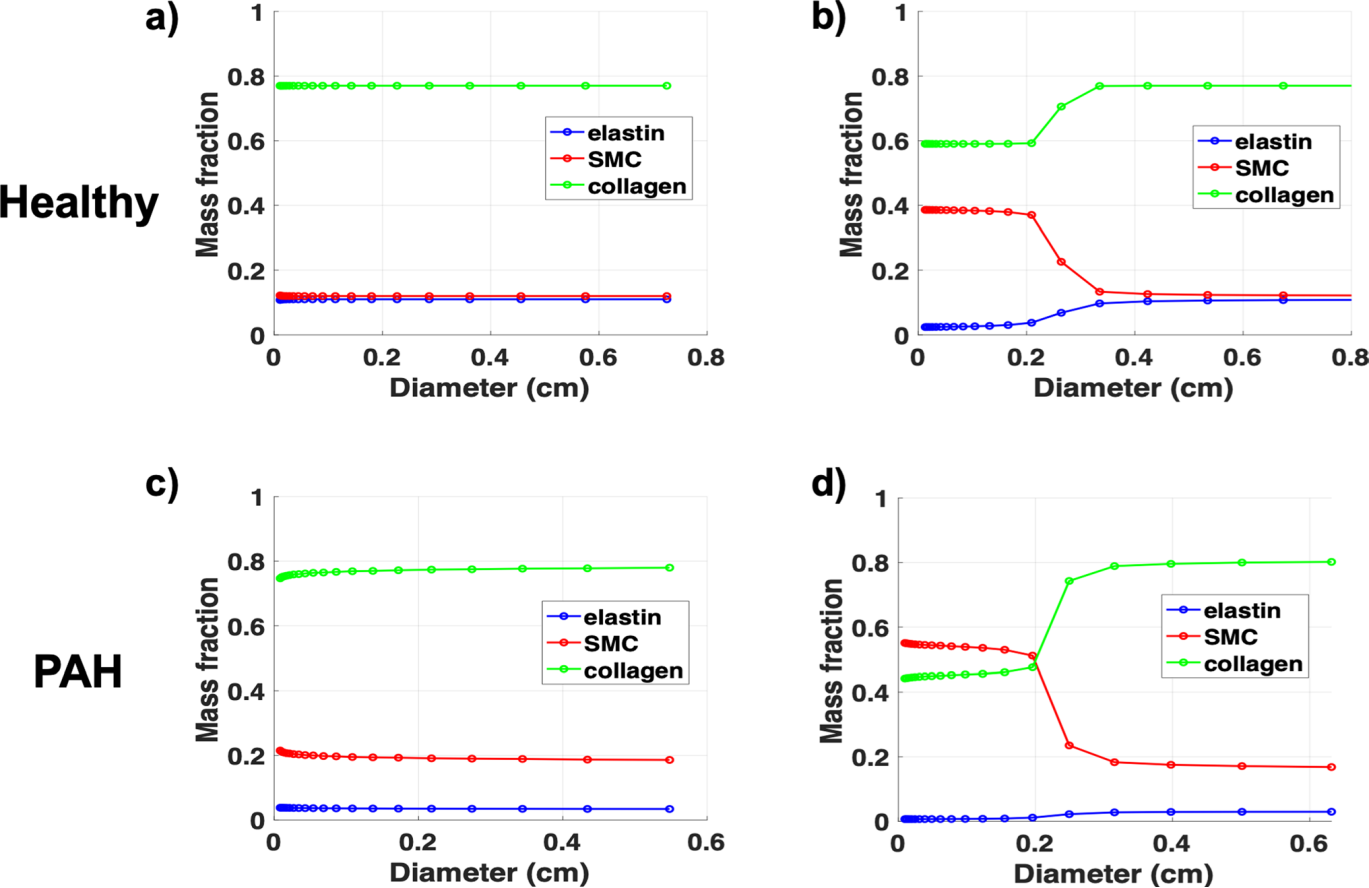
Mass fractions of constant and variable mass fractions across different diameters for (**a**) & (**b**) healthy and (**c**) & (**d**) PAH

For the healthy pulmonary vasculature, Gharahi et al. (2023) constructed the model with 2 cases on the constituents of the arterial wall and made estimates/assumptions based on the literature. For the first case of assuming a constant mass fraction, the intimal layer thickness and its constituents are neglected, as it contributes to < 15% of total thickness (Chazova et al. 1995), and the adventitial layer was assumed to be comprised of 95% collagen and 5% of elastin, neglecting other constituents such as fibroblasts and the endothelial layer for modeling simplicity. Combined with these assumptions and reported mass fractions of the constituents in the medial layer as reported by Mackay and colleagues Mackay’ et al. 1978), the constant mass fractions for a healthy vasculature as shown in Fig. 4a are defined.

However, various pulmonary artery pathology studies (Elliott and Reid 1965; Chazova et al. 1995; Townsley 2012) have shown that the mass fractions of wall constituents vary from the main pulmonary arteries to capillary arteries, transitioning from elastic to transitional, muscular, partially muscular, and non-muscular arteries. This study however excludes arterial vessels with a smaller diameter of less than 0.01 cm, instead focusing on the region of large and small vasculature in the transition between elastic and muscular arteries as highlighted in Fig. 4b. To establish the mass fractions of a PAH arterial tree, the following assumptions are made: 1) The mass of elastin (M_e_) is assumed to be the same as that in a healthy arterial wall for the corresponding generation, 2) the change in medial layer thickness is assumed to result entirely from an increase in the mass of smooth muscle cells (M_smc_). The second assumption is motivated by the study (Tobal et al. 2021). Along with the above assumptions and histological data on change in wall thickness for PAH (Chazova et al. 1995), mass fractions for the PAH arterial wall are established for both cases, as shown in Fig. 4c, d.

#### Hemodynamics with PAH

**Fig. 5:**
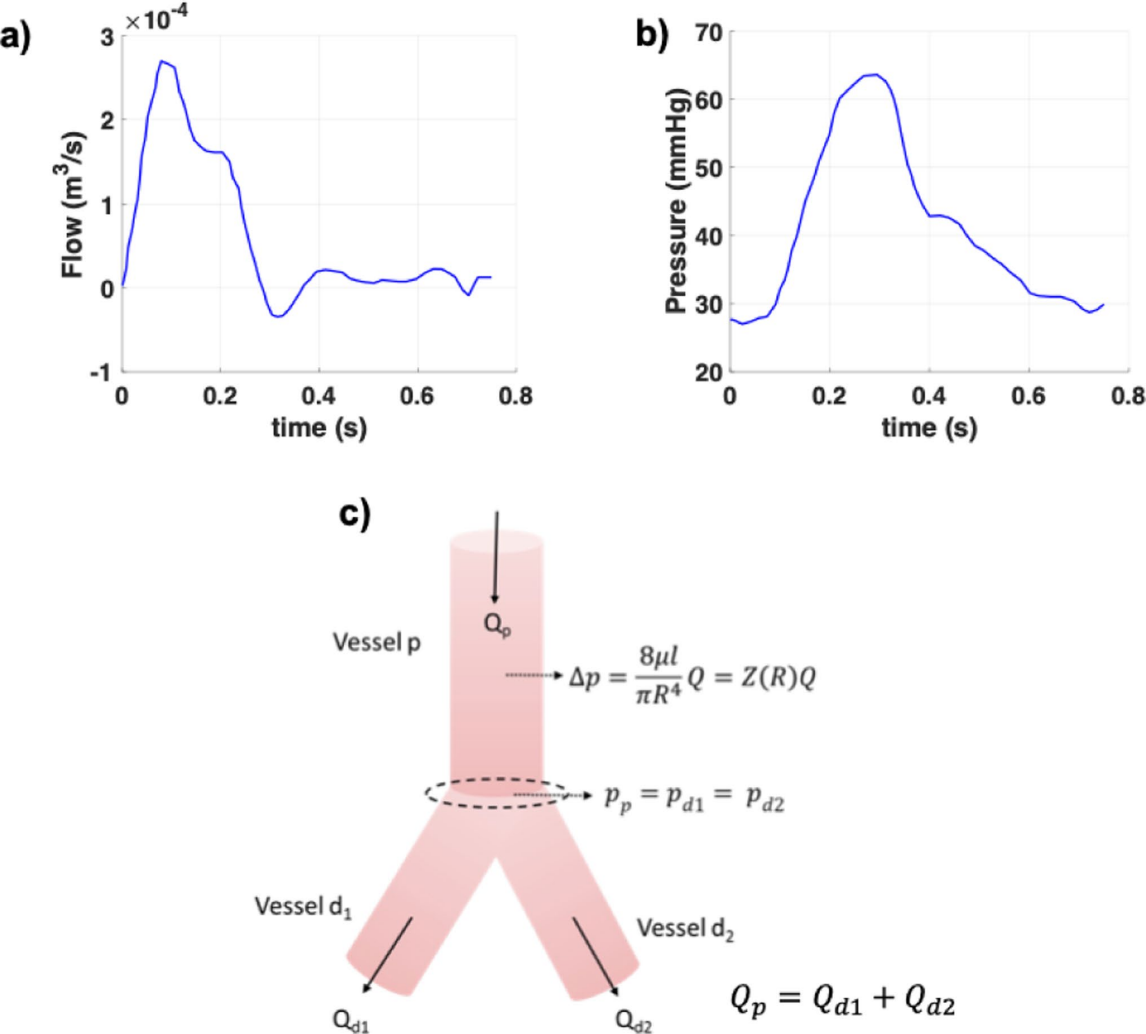
**a** Flow profile and (**b)** pressure profile of a PAH patient as reported in (Lankhaar et al. 2006), **c** hemodynamics in an arterial vessel bifurcation

In parallel with the geometric and mass fraction model construction, the model incorporates the hemodynamic aspects specific to PAH as described in (Lankhaar et al. 2006; Zambrano et al. 2018). Hemodynamic simulations within the computational model aim to replicate the dynamic conditions within PAH-affected pulmonary arteries. Along with slow time hemodynamics (minutes), pulsatile hemodynamics are evaluated using the fluid solid growth (FSG) framework and Womersley’s solution (Figueroa et al. 2009; Filonova et al. 2020; Gharahi et al. 2023) as briefly described in **Supplementary Materials**. From the PAH hemodynamics data, the inlet flow and pressure are specified at the boundaries and used to solve the hemodynamics, conserving flow, and maintaining constant pressure at each bifurcation as shown in Fig. 5c.

Using the pressure and flow profiles of the main pulmonary artery (MPA) from hemodynamic measurements of a PAH patient (Lankhaar et al. 2006), as shown in Fig. 5a, b we extend these to the 1 st generation (third bifurcation from the MPA) in our model with the following assumptions: (1) The pressure drop is negligible across the first three bifurcations, so the input pressure for the 1 st generation is the mean pressure from the MPA profile; and (2) flow is halved at each bifurcation in a symmetric tree, making the input flow for the 1 st generation 1/8th of the MPA mean flow. After defining the input flow and pressure at the 1 st generation of the arterial tree, the pressure, flow, and resistance across all subsequent generations can be evaluated using the following relations:

6 $${\mathrm{Res}} = \frac{8\mu L}{{\pi R^{4} }},$$

7 $$Q_{d1} = Q_{d2} = \frac{{Q_{p} }}{2},$$

8 $$\Delta P = {\mathrm{Res}}*Q = \frac{8\mu L}{{\pi R^{4} }}Q,$$

where *Res* is the resistance, *R* is the radius of the blood vessel, *Q* is the blood flow, and $\mu$ is the dynamic viscosity of blood, taken as 0.0035 Pa s.

### Metabolic cost of PAH arterial tree

This section explains how metabolic cost and energy are calculated, given the arterial geometry, hemodynamics, and mass fractions, in contrast to the previous study (Gharahi et al. 2023), where metabolic costs were defined from experimental data, and geometry was computed using the metabolic energy cost optimization method. Based on Gharahi et al. (2023), the total metabolic energy cost (*C*) of the pulmonary vasculature per unit length for an individual blood vessel can be defined and calculated as.

9 $$C = C_{blood} + C_{drag} + C_{wall} ,$$

10 $$C = \vartheta^{blood} \pi R^{2} + \frac{{8\mu Q^{2} }}{{R^{4} }} + \frac{2\pi R}{{\rho_{solid} }}\mathop \sum \limits_{i} \vartheta^{i} M_{R}^{i} ,$$

where ${\vartheta }^{blood}$ is the metabolic energy consumption of blood supply which is 51.7 W/m^3^ (Liu and Kassab 2007), ${\vartheta }^{i}$ is the metabolic energy consumption of vessel wall constituent *i*, which is considered to be 0 W/m^3^ for elastin, 1500 *W/m*^*3*^ for collagen and smooth muscle cells maintenance, and 0.00872 s^−1^ for active tension (Liu and Kassab 2007; Paulin and Michelakis 2014). Using the above relations, we calculate the total metabolic energy cost based on the two parallel conjectures: 1) calculating the energy cost *C* per unit length while keeping the metabolic energy consumption of the constituents ${\vartheta }^{i}$ per unit volume the same as in the healthy arterial wall and 2) calculating the energy consumption of the constituents ${\vartheta }^{i}$ for each arterial generation by adjusting the energy consumption ${\vartheta }^{i}$, while the total energy cost *C* per unit length is assumed to be same as a healthy vessel. Hemodynamic quantities were evaluated using the fluid solid growth (FSG)-based formulation introduced in our previous work (Gharahi et al. 2023). In this study, the FSG framework is used to compute wall stress and shear stress distributions for the steady-state configuration (slow timescale) without explicitly solving for temporal growth or remodeling, which will be addressed in follow-up work.

We represent the maintenance cost of smooth muscle tone with a first-order rate constant ${\alpha}_{\mathrm{act}}$(s^−1^) acting on the active specific energy density ${\psi}_{\mathrm{act}}$($J {m}^{-3}$). The product ${\alpha}_{\mathrm{act}}{\psi}_{\mathrm{act}}$ has units of $W {m}^{-3}$ and contributes to the metabolic power density of the wall. The per-length energy cost follows by multiplying by wall volume per unit length, ${\mathcal{V}}^{^{\prime}}\approx 2\pi R{\hspace{0.17em}}h$:

11 $$\underset{{\text{W m}}^{-1}}{\underbrace{{\dot{E}}_{\mathrm{act}}}}{\hspace{0.25em}\hspace{0.05em}}={\hspace{0.25em}\hspace{0.05em}}\underset{{\mathrm{s}}^{-1}}{\underbrace{{\alpha}_{\mathrm{act}}}}{\hspace{0.25em}\hspace{0.05em}}\underset{{\text{J m}}^{-3}}{\underbrace{{\psi}_{\mathrm{act}}}}{\hspace{0.25em}\hspace{0.05em}}\underset{{\mathrm{m}}^{2}}{\underbrace{(2\pi R{\hspace{0.17em}}h)}}\Rightarrow {\text{W m}}^{-1}$$

The modeling framework can be interpreted as a factorial design involving three primary factors: (1) wall composition (constant versus variable mass fractions), (2) metabolic cost assumption (constant metabolic consumption per unit volume versus variable metabolic consumption constrained by total energy expenditure), and (3) disease state (healthy versus PAH). This structure enables systematic comparison of how arterial composition and metabolic assumptions influence predicted mechanical and energetic behavior under both physiological and pathological conditions.

## Results

Using the presented data-driven computational model, key mechanical parameters and hemodynamic variables are predicted based on two scenarios with symmetric constant and variable mass fractions and compared for healthy and PAH-affected pulmonary arteries. This comparative analysis highlights the deviations in mass distribution, emphasizing the impact of PAH-induced geometric and hemodynamic changes. Understanding the differences in mass fractions provides valuable insights into the disease progression and aids in the identification of key parameters influencing PAH.

### Arterial wall mechanics

**Fig. 6:**
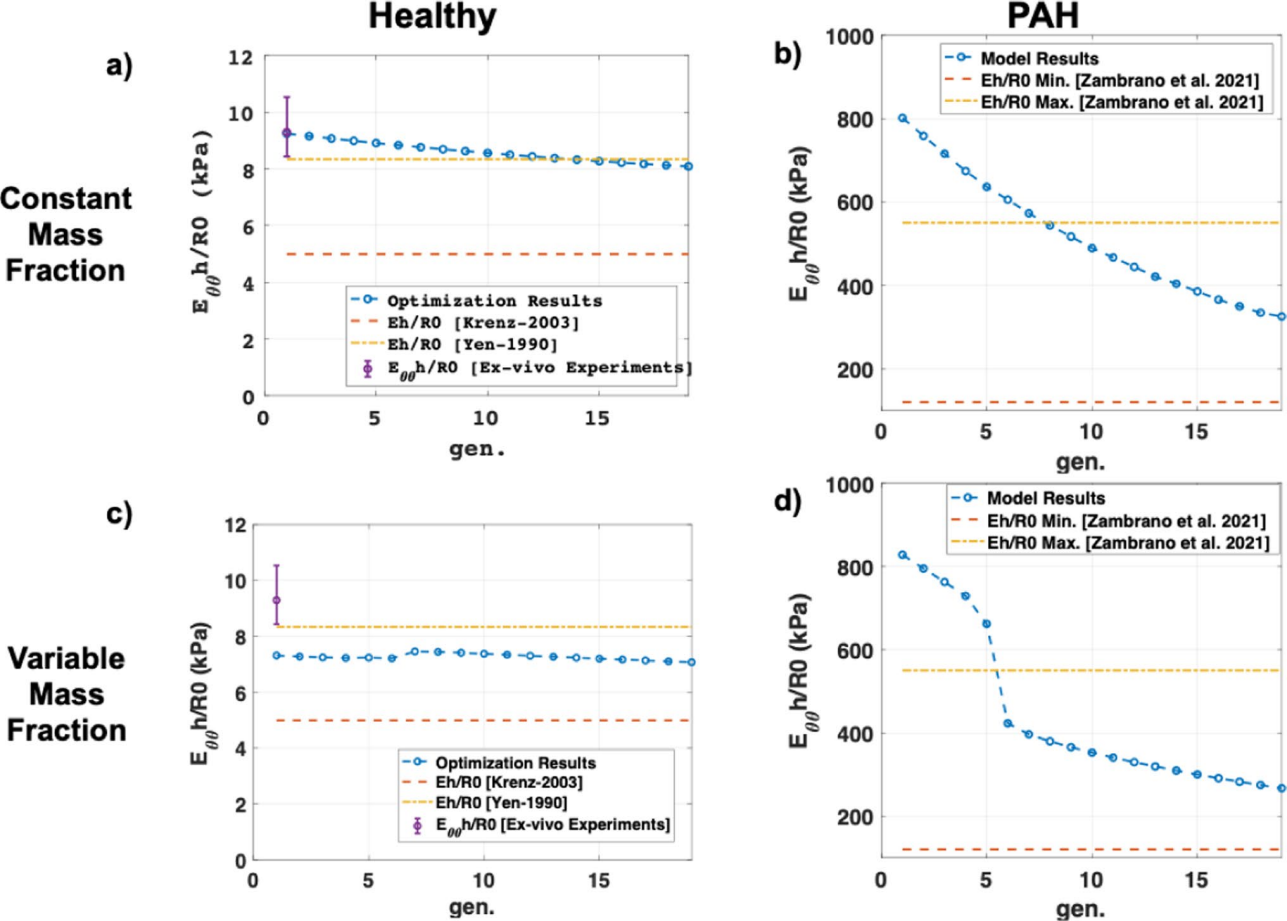
Arterial wall stiffness across different generations for healthy and PAH cases with constant and variable mass fractions compared with the literature data. Ex vivo experiments were the values computed from (Yen et al. 1990; Krenz and Dawson 2003; Zambrano et al. 2021; Wang et al. 2021)

The linearized Young’s modulus reported in Figs. 6 and 8 is obtained from the constrained mixture formulation of the arterial wall mechanics. Specifically, the tangent modulus is calculated by linearizing the strain-energy function of the composite wall around the homeostatic configuration, accounting for the mechanical contributions and mass fractions of elastin, collagen, and smooth muscle cells. This procedure yields the effective stiffness of the arterial wall used for comparison between healthy and PAH conditions.

The results of computational analysis of vascular arterial mechanical and slow time hemodynamics (mean flow and pressure) parameters are presented for arterial stiffness, wall shear stress, and linearized modulus (Baek et al. 2007; Gharahi et al. 2023). The comparison between the stiffness of healthy arteries and PAH arteries (variable stiffness case) shows a drastic increase in PAH. In Fig. 6a and c, the stiffness for the healthy case remains around 7–10 kPa across generations, aligning with previous experimental values (Krenz and Dawson 2003).

In contrast, in the PAH case Fig. 6b and d, stiffness is significantly higher, starting around 800 kPa and decreasing to approximately 300 kPa across generations—roughly 40 to 85 times higher than in the healthy condition. This stark increase illustrates the heightened arterial stiffness in PAH due to vascular remodeling and loss of compliance.

To better interpret these stiffness changes, the ratio of vessel diameter to wall thickness (D/h) across generations was evaluated (Supplementary Figure S1). The results show a pronounced reduction in D/h for PAH arteries, especially in distal generations, consistent with reported histological findings of wall hypertrophy and luminal narrowing.

**Fig. 7:**
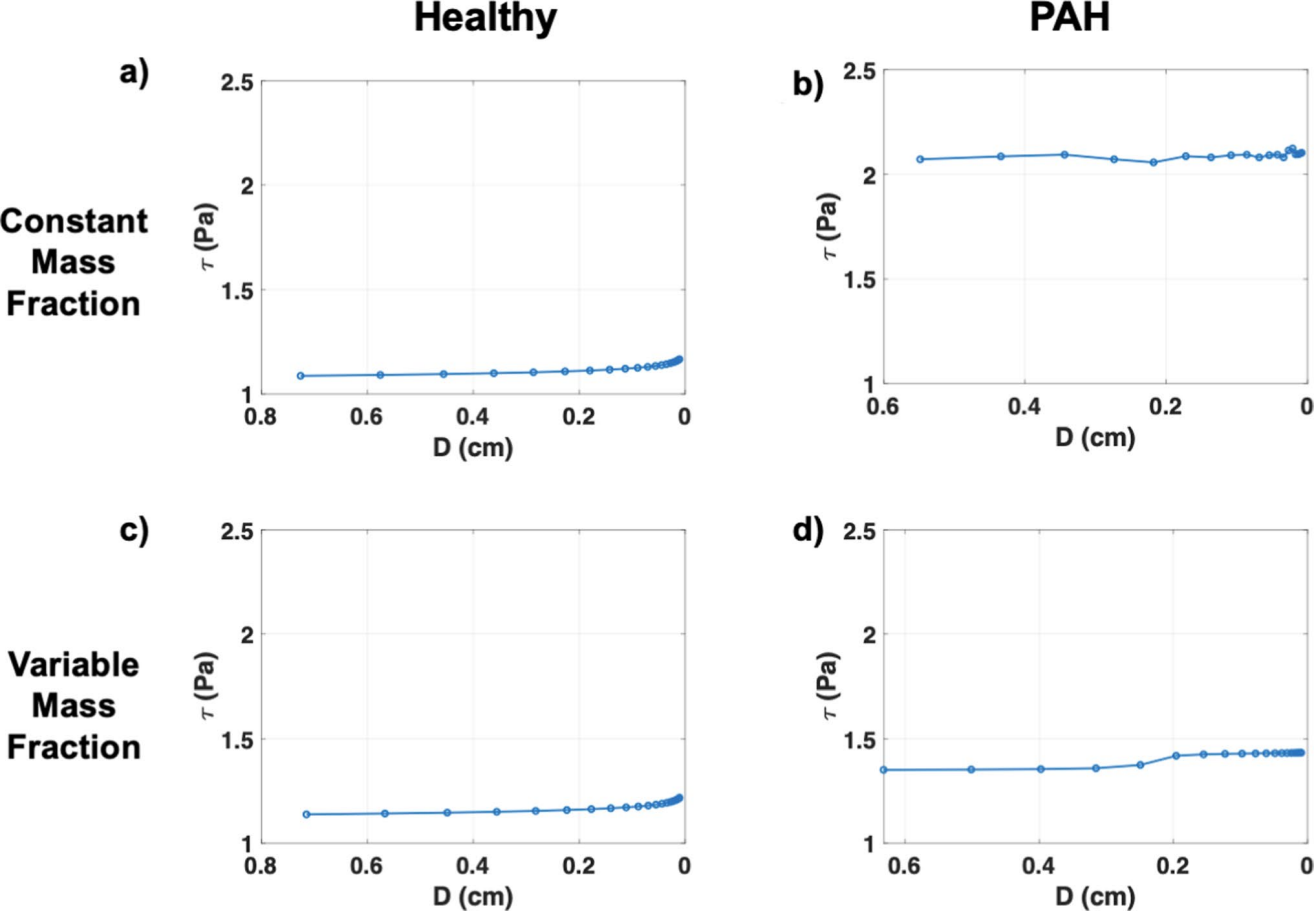
Shear stress across different generations for healthy and PAH cases with constant and variable mass fractions

In Fig. 7, the wall shear stress (WSS) $\tau$ is plotted against vessel outer diameter (D) for both healthy and PAH conditions. In Fig. 7a (healthy, constant mass fraction), shear stress starts at approximately 1.2 Pa for larger diameters and increases to around 2.0 Pa for smaller diameters. Similarly, in Fig. 7c (healthy, variable mass fraction), the trend is consistent with a slight rise in shear stress at smaller diameters.

For PAH, as shown in Fig. 7b (constant mass fraction), shear stress is notably higher and more stable, starting around 2.0 Pa across all diameters, while in Fig. 7d (variable mass fraction), it shows a more gradual increase from about 1.3 Pa to 1.5 Pa as the diameter decreases. In the symmetric tree considered here, PAH remodeling results in a relatively uniform WSS distribution across generations due to reduced geometric variability. However, this outcome reflects an effect of the idealized, symmetric architecture rather than a physiological prediction. In realistic pulmonary arterial trees, asymmetric branching and local morphometric variations contribute to significant WSS heterogeneity (Zamir 1999a; Yang et al. 2019). In PAH conditions, as shown in Fig. 7b and d, the elevated mean pressure reflects increased vascular resistance and geometric narrowing of the proximal arteries, which together yield higher flow velocity gradients and consequently higher WSS. Thus, the rise in WSS is primarily a result of luminal narrowing and altered flow distribution, rather than a direct effect of pressure.

**Fig. 8:**
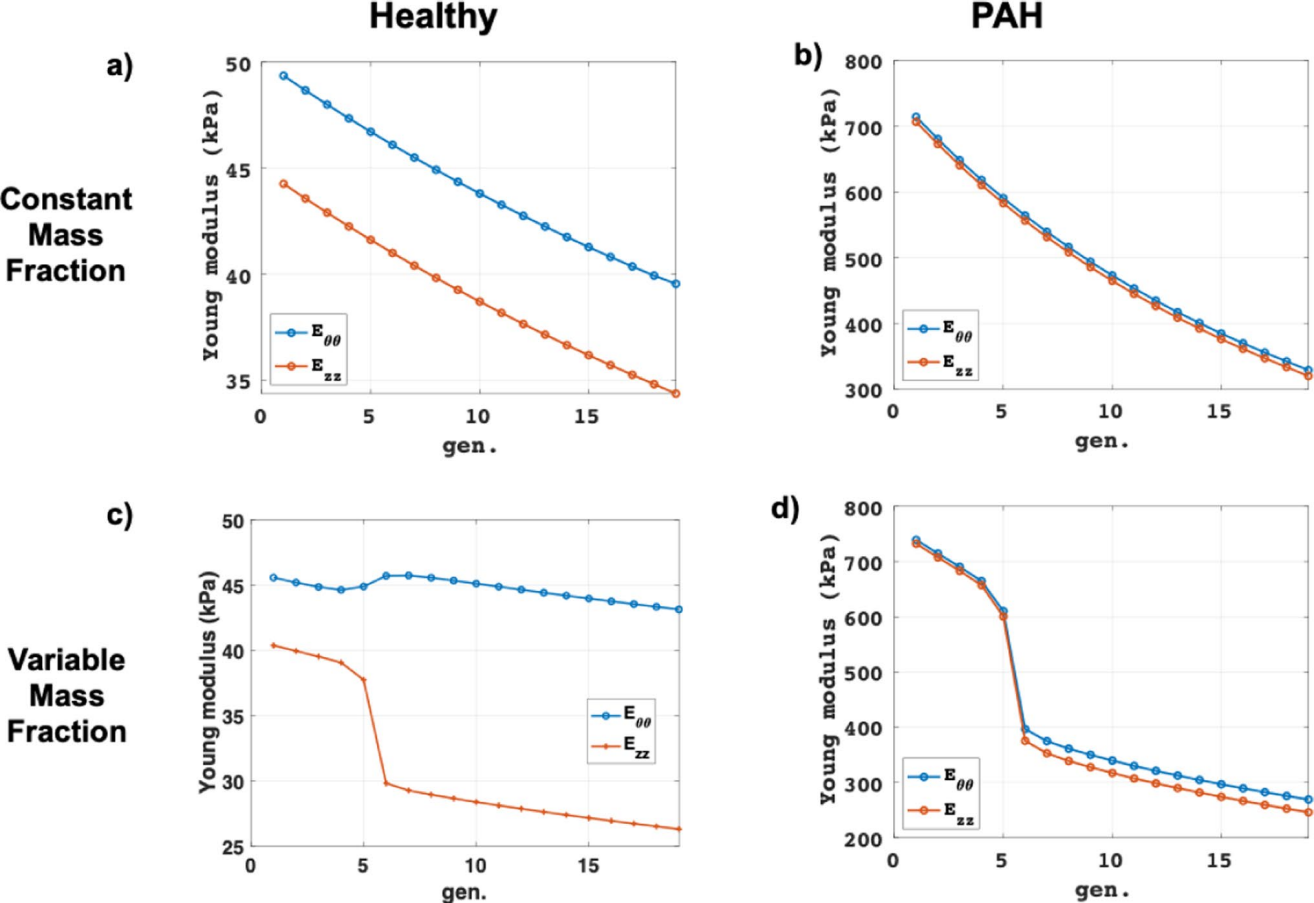
Young’s modulus across different generations for healthy and PAH cases with constant and variable mass fractions

In Fig. 8, the graphs compare the (linearized) Young’s modulus across generations for both healthy and PAH conditions. In Fig. 8a (for a healthy subject and constant mass fraction), the modulus starts around 50 kPa and gradually decreases to approximately 35 kPa in the later generations. In Fig. 8b (PAH, constant mass fraction), the modulus is significantly higher, starting at approximately 700 kPa and declining steadily. In Fig. 8c, d (PAH with variable mass fraction), the modulus exhibits a distinct drop between generations 5 and 6, highlighting the drastic stiffening and mechanical alterations in the PAH vasculature.

### Pulsatile hemodynamics using Womersley’s solution: healthy versus PAH

**Fig. 9:**
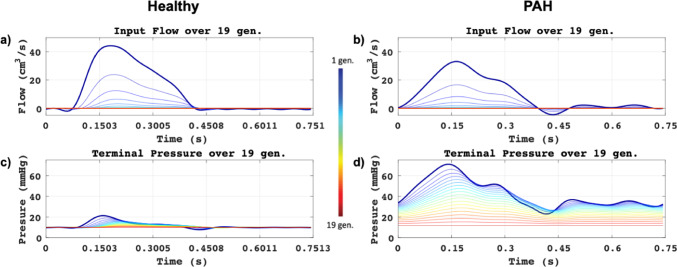
Pulsatile hemodynamics of healthy and PAH vasculature (**a)** & (**b**) flow profile and (**c**) & (**d**) pressure profile

Pulsatile (fast-time) hemodynamics were evaluated for both healthy and PAH vasculature using a symmetric arterial tree framework with constant mass fractions. Figure 9 shows the pressure and flow waveforms across all generations of the pulmonary arterial tree. In the healthy case, the inlet waveform is prescribed from published clinical flow and pressure data (Zambrano et al. 2018), and the computed waveforms demonstrate a physiologic progressive reduction in flow amplitude and a steady decrease in mean pressure from proximal to distal vessels, consistent with reported measurements of pulmonary circulation behavior (Evans et al. 2021; Gharahi et al. 2023). Under PAH conditions, the waveforms exhibit marked alterations: Flow becomes more irregular with a noticeable reversed flow component around t ≈ 0.5 s, and distal generation amplitudes are substantially reduced. Pressure remains elevated across the arterial tree and shows limited pulsatility, reflecting increased vascular stiffness and reduced compliance due to pathological remodeling. These features align with clinical observations of impaired pulsatile dynamics in PAH.

### Metabolic energy cost of the arterial segment

**Fig. 10:**
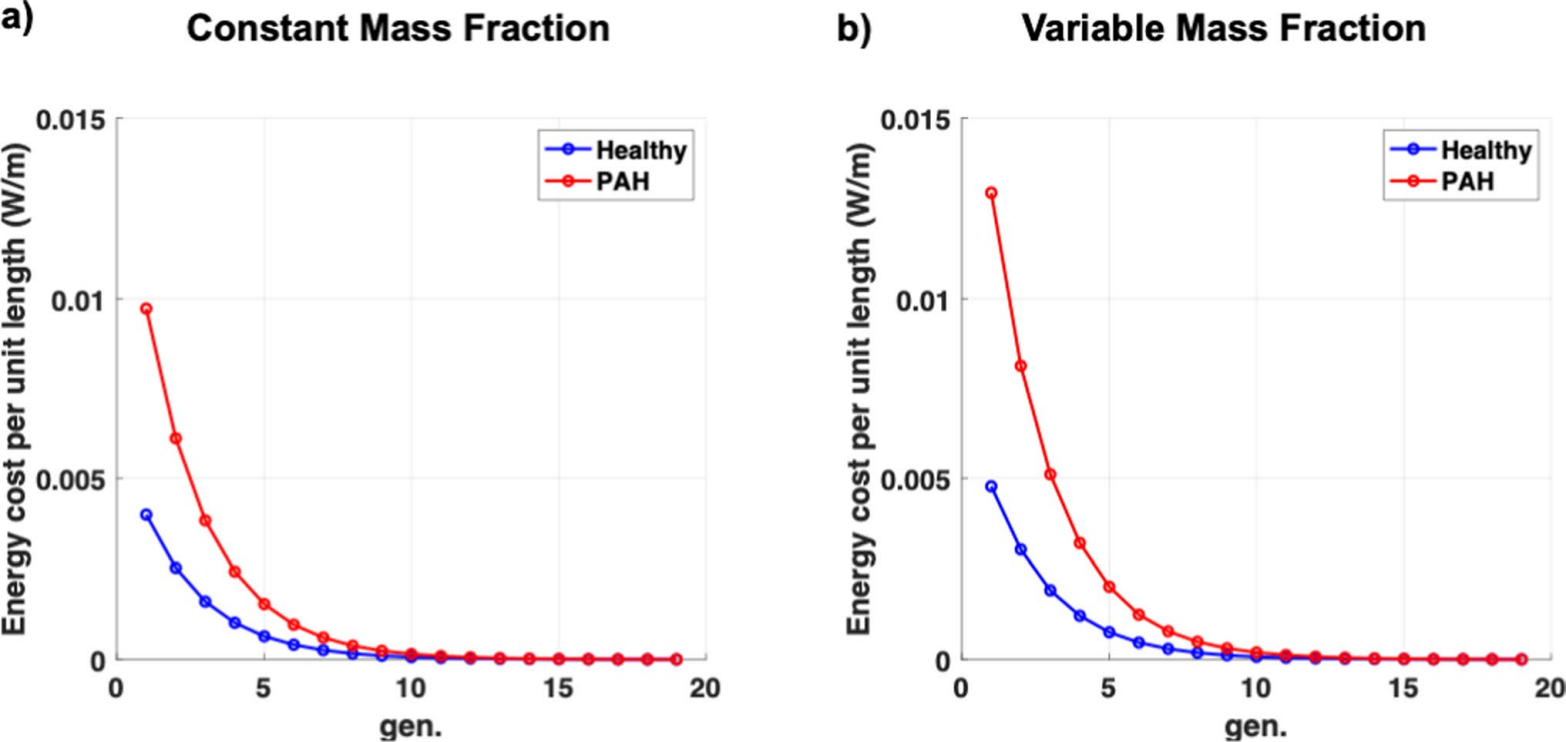
Energy cost per unit length across different generations of the arterial tree for healthy and PAH vasculature for (**a**) constant and (**b**) variable mass fractions

The metabolic energy cost for the arterial segment is computed for the two cases of constituent mass fractions (constant vs. variable) for both healthy and PAH vasculature, assuming a constant metabolic energy cost per unit volume for arterial constituents per unit volume (the first conjecture), plotted in Fig. 10a, b. For the constant constituent mass fraction case, where the metabolic energy consumption of the constituents ${\vartheta }^{i}$, same as healthy arterial wall, the metabolic energy cost per unit length is plotted against the generation numbers for the PAH and healthy subject. It is computed at approximately 0.01 W/m for unit length in the first generation and gradually decreases across the subsequent generations, while for the healthy vasculature, it begins at around 0.004 W/m in the first generation. The computed results show that the energy cost in the PAH case is more than twice that of the healthy case, especially in the initial generations.

**Fig. 11:**
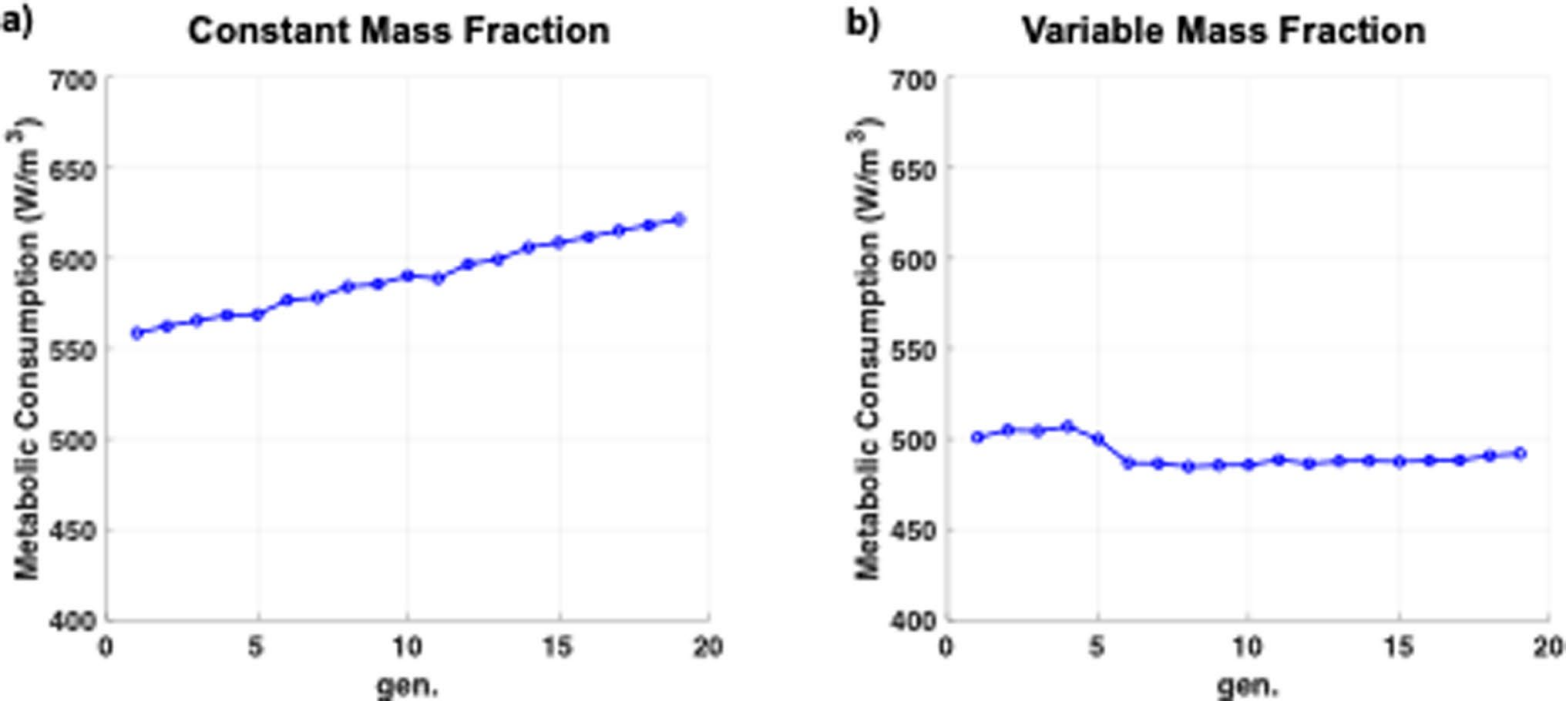
Metabolic consumption of the arterial wall across different generations of the arterial tree when both healthy and PAH models are constrained to share the same total energy cost per unit length for (**a**) constant and (**b**) variable mass fractions. The metabolic consumption is expressed in W/m^3^ (per unit vessel wall volume), which differs from Fig. 10 where energy cost is shown in W/m (per unit length), enabling direct comparison of metabolic requirements between healthy and PAH conditions

In contrast, for the second conjecture as shown in Fig. 11, the metabolic cost is evaluated assuming that the energy cost per unit length is the same as that of the healthy case. In the case of constant mass fraction, the metabolic consumption increases linearly across generations, starting at approximately 500 W/m^3^ and rising to about 625 W/m^3^. For the arterial tree with variable mass fraction, the metabolic consumption remains relatively stable across generations, fluctuating around 450–500 W/m^3^. This indicates that reducing metabolic consumption makes overall energy expenditure comparable to that of healthy arteries.

## Discussion

A computational vascular model should accurately capture physiologically realistic tree morphometry, as it affects vascular resistance and impacts long-term vascular adaptation and hemodynamics during the cardiac cycle. However, creating a complete and physiologically precise morphometry of the distal pulmonary arterial tree is challenging due to limitations in data acquisition and modeling complexity (Burrowes et al. 2005). Inspired by Murray’s law, which optimizes metabolic energy cost in biological systems and helps identify bifurcation patterns (Murray 1926), several approaches have been proposed to characterize vascular network architecture. These include fractal-based morphometry (Zamir 1999b; Kamiya and Takahashi 2007; Ionescu et al. 2009) and multiscale hemodynamic and fluid–solid interaction (FSI) analyses (Olufsen et al. 2000; Mittal et al. 2005; Van De Vosse and Stergiopulos 2011; Qureshi et al. 2014). Nevertheless, these studies often rely on simplified vessel wall representations that do not account for the contributions of individual structural constituents—an essential factor in understanding structural changes associated with PAH. To address this limitation, Gharahi et al. (Gharahi et al. 2020, 2023) developed computational models that enable long-term hemodynamic and fast hemodynamics with FSI analysis, integrating physiologically realistic tree morphometry and structurally motivated vessel wall models to estimate healthy homeostatic stresses and hemodynamic variables.

Building upon Gharahi’s framework, this study presented a baseline model of PAH vasculature, defining key geometric, mechanical, and hemodynamic variables while estimating the metabolic characteristics of affected arteries. It also compares critical parameters—such as arterial stiffness, wall shear stress, and energy costs—between healthy and PAH-affected arteries. This hybrid (data-driven and computational) baseline model accurately reflects key morphometric changes observed during disease progression. By incorporating variations in wall thickness, lumen narrowing, and hemodynamic parameters, the model enables a detailed analysis of disease-specific mechanical and structural properties of the pulmonary vasculature. Establishing baselines for both healthy and diseased pulmonary arteries reveals significant differences in geometric, mechanical, and hemodynamic variables.

The first key result of data-driven computational model is that arterial stiffness in PAH-affected vessels is 40 times higher than in healthy arteries, indicating a severe loss of elasticity. The computational results align with these observations, such as reported structural stiffness (${E}_{\theta \theta }h/{R}_{0}$) 7.1 to 9.3 kPa for healthy subjects (Yen et al. 1990; Krenz and Dawson 2003) and in the range of 280 to 840 kPa in PAH patients (Zambrano et al. 2021) shown in Fig. 6, demonstrating a comparable magnitude for the Young's modulus across arterial generations. The computed axial and circumferential moduli exhibit trends consistent with experimental data, showing increased stiffness in proximal vessels, which decrease progressively along the distal pulmonary arterial tree. Other studies on pulmonary arteries in PAH-afflicted calves reported that the Young’s modulus under PAH conditions was approximately three to four times higher than in normal arteries, with values ranging from 100 to 200 kPa—substantially exceeding those in healthy pulmonary arteries with the values ranging from 50 to 100 kPa (Lammers et al. 2008). These findings align with calf models of PAH, where increased vascular stiffness is a hallmark of disease-induced remodeling. For example, mean elastic modulus values in PAH conditions have been reported as 192 ± 56.7 kPa, compared to 97 ± 30.1 kPa in healthy calf controls (Hunter et al. 2010a). However, in the calf model, elastin is the ECM protein primarily responsible for vessel remodeling and vascular elasticity, which may explain why vessel stiffness is only mildly increased in the calf PAH model compared with human PAH.

Besides the increased stiffening, it is worth noting that in healthy vasculature the divergence between circumferential and axial moduli in the healthy vasculature reflects the greater role of elastin in bearing physiological pressure loads, resulting in more independent mechanical responses in the circumferential and axial directions. In contrast, the PAH condition shows strong coupling of ${E}_{\theta \theta }$ and ${E}_{zz}$ due to substantial collagen accumulation, increased SMC content, and earlier recruitment of collagen fibers under elevated wall stress. These changes shift load-bearing responsibility away from elastin toward structurally aligned collagen bundles, producing stiffness increases in both directions simultaneously. This results in the abrupt rise and tighter coupling observed in generations 5–6 in Fig. 8c–d, where mechanical remodeling becomes dominant.

Secondly, a difference in WSS distribution is observed between healthy and PAH-affected arteries. WSS plays a crucial role in normal physiological mechano-sensing and is linked to pathological signaling and disease progression (Wang and Valdez-Jasso 2021; Allen et al. 2023). For healthy pulmonary arteries, the results in Fig. 7a and c indicate that WSS increases as vessel diameter decreases, a trend consistent with normal hemodynamic behavior and flow distribution in arterial trees. The presence of constant or variable mass fractions does not significantly alter this gradient under healthy conditions. Such findings align with prior studies emphasizing the physiological role of WSS in maintaining endothelial homeostasis and nitric oxide (NO) production, which collectively ensure vascular stability. Under PAH conditions, as shown in Fig. 7b and d, elevated pressures in the proximal pulmonary arteries lead to an overall increase in baseline WSS compared to healthy arteries. The elevation in WSS observed in Fig. 7b, d arises primarily from the reduced internal diameter in PAH, consistent with the scaling $\uptau \sim \mathrm{Q}/{\mathrm{R}}^{3}$ assuming for a comparable total pulmonary blood flow in healthy and PAH subject. The WSS values reported correspond to the slow timescale homeostatic model, and direct comparison of pulsatile WSS is not included due to the limited availability of validated experimental datasets.

The increase in WSS observed in our computational simulations is consistent with computationally estimated results by (Yang et al. 2019; Szafron et al. 2024), who reported elevated WSS in pathological vascular remodeling, further supporting the concept that mechanical forces significantly influence vascular adaptation in PAH. In smaller, distal vessels, heightened WSS may exacerbate endothelial injury and trigger inflammatory signaling, promoting pathological changes such as intimal hyperplasia and medial thickening (Humbert et al. 2019). In contrast, larger, proximal arteries in PAH cases experience reduced WSS, contributing to endothelial dysfunction through impaired NO production and upregulation of pro-inflammatory mediators. This dual pattern, increased WSS in distal arteries and decreased WSS in proximal arteries, highlights the complexity of hemodynamic forces driving disease progression (Tang et al. 2012). The variability of WSS across the pulmonary arterial tree, as depicted in the computational model, aligns with advanced imaging studies such as those utilizing four-dimensional flow MRI (Barker et al. 2015) (0.4 Pa (control) vs. 0.22 Pa (PH) at the main pulmonary artery). From a therapeutic perspective, normalizing WSS presents a promising strategy to mitigate PAH progression. Pharmacological interventions such as PDE5 inhibitors and prostacyclin analogs (Tettey et al. 2021), which reduce pulmonary arterial pressures, may indirectly restore WSS to more physiological levels, thereby improving endothelial function. Emerging treatments like Sotatercept, which target structural remodeling (Doggrell 2023), may further enhance the normalization of WSS.

Lastly, adopting the parallel conjectures allows for a direct comparison of metabolic cost and the implications for energy efficiency. Under the first conjecture, where metabolic energy cost per unit volume remains fixed, calculations show that the total metabolic energy expenditure per unit length effectively doubles. In contrast, the second conjecture assumes variable metabolic consumption per unit volume, resulting in a substantial decrease in vessel wall metabolic demand when the total energy cost per unit length is held constant and comparable to healthy vessels. Maintaining constant metabolic consumption per unit volume would lead to excessive overall energy expenditure, potentially imposing significant physiological stress; thus, reducing metabolic demand per unit volume appears beneficial from an energy efficiency perspective. Specifically, for an arterial tree model with variable mass fraction, metabolic consumption per unit length stabilizes around 450–500 W/m^3^ across generations, as illustrated in Fig. 11b. This suggests that moderating wall metabolic consumption helps align total energy expenditure with that of healthy arteries. Consequently, the scenario described by the first conjecture is likely unrealistic, especially given the reduced energy efficiency and glycolytic shift observed in PAH (Peng et al. 2016), thereby lending support to the second conjecture, which posits metabolic adaptation through redistribution of mass and energy expenditure.

While the Warburg effect was primarily studied in cancer research (Benny et al. 2020), by the early 2010 s, the heightened activity of pyruvate dehydrogenase kinase (PDK) was implicated in cardiac hypertrophy (Piao et al. 2010), potentially leading to heart failure, broadening the scope of the Warburg phenomenon to other conditions (Dabral et al. 2019; Huang et al. 2022). The role of hypoxia signaling gained prominence in both non-malignant and malignant hyperproliferative vascular diseases like pulmonary hypertension, atherosclerosis, and vascular restenosis. It has been suggested that metabolism is modified in stressed conditions such as hypoxia, oxidative stress, and inflammation, being altered from the usual mitochondrial oxidative phosphorylation to less efficient glycolysis (Piao et al. 2010; Dabral et al. 2019; Benny et al. 2020; Huang et al. 2022). Especially, it has been suggested that a key feature of PAH is the reduced contractility of PASMCs under hypoxic conditions, largely driven by mitochondrial dysfunction. In PASMCs isolated from PAH patients, the expression of mitochondrial ETC components I–V is significantly reduced, leading to decreased mitochondrial oxygen consumption (Shi et al. 2020). In animal models, the activity of mitochondrial respiratory chain complexes I–III is reduced by approximately 50%, correlating with a similar reduction in overall mitochondrial oxygen consumption (Shi et al. 2020). This can be corroborated by the reduction in metabolic consumption as shown in Fig. 11. This dysfunction in mitochondrial oxidative phosphorylation directly impacts PASMC contractility by limiting ATP production, which is critical for muscle function. Reduced energy availability under hypoxia leads to diminished contractility and promotes the onset of vascular remodeling characteristics of PAH.

During the early stages of PAH, PASMCs exposed to chronic hemodynamic stress exhibit further declines in mitochondrial oxidation and endothelial cell activity, exacerbating the disease’s pathophysiology. The energy deficit caused by mitochondrial impairment not only weakens contractility but also drives PASMC proliferation, contributing to arterial thickening and increased pulmonary vascular resistance (Marshall et al. 2018). Hypoxia plays a pivotal role in regulating these metabolic shifts, pushing PASMCs toward a glycolytic phenotype that further disrupts their normal contractile function (Taggart and Wray 1998). Mitochondrial dysfunction in PAH also involves hyperpolarized mitochondria, which suppress apoptosis and promote cell proliferation. This is accompanied by reduced production of mitochondrial-derived reactive oxygen species (mROS) and activation of hypoxia-inducible factor (HIF)−1α, which stimulates glycolysis (Ghofrani et al. 2011; Xu et al. 2021).

Beyond glucose metabolism, the metabolic alterations in PAH extend to lipid and amino acid metabolism. Non-oxidized sugars, lipids, and amino acids are redirected toward biosynthetic pathways, which support cellular proliferation and survival (Paulin and Michelakis 2014); this altered metabolic state contributes to the resistance to apoptosis, a hallmark of PAH, and promotes the extensive vascular remodeling observed in the disease. Recent studies also suggest that these metabolic changes activate epigenetic mechanisms and inflammation via mitochondria-based inflammasomes, linking metabolic dysfunction to the progression of vascular remodeling (Xu et al. 2021).

The significance of this model lies in its potential as a platform for future integration of pharmacological pathways and treatment simulations. While this study focuses on establishing the baseline network, the framework is inherently designed to accommodate additional pathways, such as the nitric oxide-cGMP-PKG signaling cascade (Mullagura et al. 2021), enabling the exploration of therapeutic interventions and patient-specific treatment predictions. The ability to extend this baseline into a predictive model for treatment response offers a transformative tool for advancing the understanding of PAH progression and its management. By providing a detailed, data-driven representation of the diseased arterial network, this study bridges an important gap between theoretical modeling and experimental observations. This alignment with existing experimental and clinical data enhances the reliability of the framework and underscores its potential to serve as a reference for further studies on vascular remodeling. Additionally, this baseline model is significant for its versatility. Beyond its utility in PAH research, it serves as a foundation for broader applications in vascular diseases involving remodeling and altered hemodynamics. Its modular structure makes it adaptable for investigating interactions between biomechanics and biochemical pathways, positioning it as a critical resource for interdisciplinary studies. Overall, the established model sets the stage for integrating biochemical pathways and pharmacological simulations, paving the way for the development of predictive tools to optimize treatment strategies for PAH.

This work represents an important step forward compared to previous frameworks such as Gharahi et al. (2023), which focused primarily on healthy homeostatic optimization without incorporating metabolic energetics, disease-specific geometric remodeling, or detailed constituent-driven mechanical changes. By integrating experimentally derived mass fraction distributions, PAH-specific morphometry, and metabolic energy consumption into a unified arterial tree model, this study provides quantitative predictions such as a 40–85-fold increase in circumferential stiffness in proximal arteries and a 3–fourfold reduction in diameter-to-thickness ratio across generations that were not previously available. These findings offer a mechanistic foundation for understanding baseline PAH vascular behavior and enable future studies on treatment response modeling. While this study presents a significant step forward in modeling the baseline PAH arterial network, several limitations must be acknowledged. One limitation arises from the assumptions and idealizations made during the modeling process. For example, the geometrical and structural parameters used to represent the pulmonary arterial network are derived from limited datasets and may not capture the full variability observed across different patients and disease stages (Bartolo et al. 2022). The simplifications, while necessary to build a tractable model, may exclude some intricate features of vascular remodeling, such as loss of elastin, localized heterogeneity in wall thickening or regional variations in stiffness (Rol et al. 2017). In particular, a limitation of the present model is that the simplified morphometry and exclusion of proximal arterial pathology likely underestimate WSS compared with clinical reports, especially in large arteries, which remain an important direction for refinement. Similarly, key pathological features such as changes in endothelial cell proliferation, plexiform lesions, or severe vasoconstriction observed even in moderate or severe stages (Evans et al. 2021) were not included, which is evident in that the calculated WSS values in this study were underestimated compared with clinical and numerical studies (Edwards et al. 1998; Truong et al. 2013; Bartolo et al. 2022), even though this study did demonstrate an increasing trend in WSS for PAH. Another limitation is the gap in experimental data on metabolic costs specific to PAH. While the model incorporates hemodynamic and structural changes, the lack of comprehensive data on the metabolic energy consumption of PASMCs in diseased states prevents a deeper integration of energy dynamics into the framework. This gap restricts the ability to fully capture the energetic shifts, such as those driven by the Warburg effect, in the pathophysiology of PAH. Furthermore, the model assumes a standardized disease state, which poses challenges when addressing the heterogeneity of disease progression. The progression and severity of PAH vary significantly among patients, influenced by factors such as genetics, comorbidities, and environmental exposures (Talwar et al. 2017). This variability makes it difficult to develop a single model that encompasses all degrees of disease progression. The interplay between environmental and acquired factors and an individual’s genetic predisposition plays a significant role in the heterogeneity, variable onset, and progression of PAH. Notably, mutations in the BMPR2 gene are the most common genetic drivers of heritable and idiopathic PAH which are strongly associated with “metabolic reprogramming” (Fessel et al. 2012a; Cuthbertson et al. 2023). Cells harboring BMPR2 mutations frequently exhibit a metabolic shift characterized by increased glycolysis and mitochondrial dysfunction. Metabolomic profiling of endothelial cells with these mutations reveals upregulation of glycolysis, the pentose phosphate pathway, nucleotide salvage, and polyamine biosynthesis, alongside marked impairments in the tricarboxylic acid (TCA) cycle and fatty acid oxidation pathways (Fessel et al. 2012b). Accumulating evidence from both human clinical studies and experimental models highlights these metabolic derangements as major contributors to the diverse clinical phenotypes, disease severity, and differential treatment responses observed in PAH patients carrying BMPR2 mutations (Thenappan et al. 2018c). A computational hemodynamic-metabolic modeling approach shows promise for deepening our mechanistic understanding of how environmental triggers and genetic susceptibility converge to shape the heterogeneity, timing of disease onset, and progression patterns in PAH.

In this study, the elastin mass fraction in PAH was assumed to be similar to that of the healthy arterial wall within comparable generations. While this assumption is consistent with the low turnover and minimal metabolic cost of elastin in adult vasculature, we acknowledge that histological evidence from severe PAH reports elastin fragmentation and localized loss in distal vessels. Due to insufficient quantitative data to parameterize elastin degradation along the tree, elastin remodeling was not explicitly incorporated. Future work will include elastin turnover and degradation dynamics as additional remodeling pathways. As such, while this model serves as a robust baseline, its predictive capacity may be limited for extreme or atypical cases. Additionally, Eq. (4) assumes a fixed interstitial fluid volume fraction (ϕ_f = 0.7), which is reasonable for healthy vessels but may not hold in distal PAH where increased cellularity and inflammation alter extracellular water content. These changes would modify the solid-to-fluid ratio and could influence metabolic cost calculations, representing an important direction for future refinement. Despite these limitations, the model provides a valuable foundation for advancing PAH research. Its modular design would allow for the integration of additional data and pathways as they become available, enabling refinement and expansion over time (Savale et al. 2018). While no model can fully capture the complexity of PAH, this study marks an important step forward, providing a framework that can guide further research and facilitate the development of targeted therapies.

In conclusion, the construction of a comprehensive baseline model for PAH involves a meticulous process spanning arterial geometry, hemodynamics, and metabolic considerations. The three stages (geometry construction, pathological estimation, and metabolic costs analysis) ensure a robust representation of PAH arterial mechanics. The findings highlight the significant alterations in mechanical properties, particularly the increased stiffness and energy expenditure in PAH vasculature. These insights are crucial for advancing our understanding of PAH pathophysiology and for developing future treatment strategies. The model offers potential for simulating patient-specific arterial responses to treatments, contributing to a more effective clinical approach in managing PAH.

## Supplementary Information

Below is the link to the electronic supplementary material.

Supplementary file1 (DOCX 202 KB)

Supplementary file2 (DOCX 236 KB)

## Data Availability

Publicly available in a repository: All data and computational code supporting the findings of this study are publicly available in the GitHub repository at: https://github.com/harithavnaidu/PAH-Geometric-Remodeling.git
