# Supplementary material for: Baseline state for pulmonary vasculature with pulmonary arterial hypertension: effect of geometric remodeling and metabolic shift
Source: Biomech Model Mechanobiol. 2026 Jun 10;25(3):68. doi: 10.1007/s10237-026-02076-4 (PMC13253922; doi:10.1007/s10237-026-02076-4)
Supplement: Supplementary file 2 — Supplementary file2 (DOCX 236 KB) [file 10237_2026_2076_MOESM2_ESM.docx]

# Supplementary Material for “Baseline State for Pulmonary Vasculature with PAH: Effect of Geometric Remodeling and Metabolic Shift”

Haritha N. Mullagura^1*^, Hamidreza Gharahi^2^, C. Alberto Figueroa^2,3^, Seungik Baek^1^

^1^Department of Mechanical Engineering, Michigan State University, East Lansing, MI

^2^Section of Vascular Surgery, Department of Surgery, University of Michigan, Ann Arbor, MI

^3^Department of Biomedical Engineering, University of Michigan, Ann Arbor, MI

# Morphometry of Pulmonary Arterial Tree

The pulmonary arterial geometry is adapted from (Gharahi et al. 2023) with 19 generations and a terminal vessel diameter threshold of 50 µm. Morphometric parameters such as the number of vessels per generation and branching connectivity are preserved from the healthy baseline. In contrast, wall thickness and mass fractions of constituents (elastin, collagen, SMC) are modified to represent PAH remodeling. A detailed summary of unchanged and altered parameters is included in Table S1. The current implementation assumes symmetric daughter-to-parent area ratio (1.0). Asymmetric network generation will be explored in future work using the same metabolic energy formulations.

**Table S1. Summary of parameters between healthy and PAH baseline geometry**

| **Category** | **Parameter** | **Symbol** | **Healthy Model (Gharahi et al., 2023)** | **PAH Model Modification** | **Unit** | **Reference / Note** |
| --- | --- | --- | --- | --- | --- | --- |
| **Tree structure** | Number of generations | N | 19 | Unchanged | – | Gharahi et al., 2023 |
|  | Daughter-to-parent area ratio | – | 1.0 (Symmetric) | Unchanged | – | Gharahi et al., 2023 |
|  | Length-to-radius relation | – | L=6.2 R^1.1^ mm | Unchanged | – | Gharahi et al., 2023 |
|  | Minimum vessel diameter | Dₜ | ~50 µm (Gen 19) | Unchanged | µm | Huang et al., 1996 |
| **Wall geometry** | Wall thickness ratio | h/Dₑ | 0.07 | Data-based linear fit with using Eq. (1) and Eq. (2) | – | Gharahi et al., 2023; Rol et al., 2017 |
|  | Wall thickness (absolute) | h | Function of vessel radius | Increased 2–3× in distal vessels | µm | Rol et al., 2017 |
| **Material composition** | Elastin, collagen, SMC fractions | – | Homeostatic values | Altered for PAH remodeling | – | Rachev & Hayashi, 1999 |
| **Hemodynamics** | Blood density | ρ | 1060 | Unchanged | kg/m³ | Liu & Kassab, 2007 |
|  | Blood viscosity | μ | 0.0035 | Unchanged | Pa·s | – |
|  | Terminal pressure | Pₜ | ~10 | ~40 | mmHg | Lankhaar et al., 2006 |
|  | Input flow waveform | Q | Scaled cardiac waveform | Scaled identical waveform | mL/s | Gharahi et al., 2023 |
| **Metabolic parameters** | Metabolic energy – elastin | θₑ | 0 | 0 | W/m³ | Liu & Kassab, 2007 |
|  | Metabolic energy – collagen + SMC | θ_c/smc | 1500 | 1500 | W/m³ | Liu & Kassab, 2007 |
|  | Active tone term | θ_active | – | 0.00872 | s⁻¹ | Paulin & Michelakis, 2014 |

The D/h ratio was computed using the modeled inner diameter (2R) and wall thickness (h) for each generation. The PAH model shows a consistently lower D/h ratio than the healthy baseline, indicating increased wall thickening and reduced lumen diameter toward distal generations.

**Figure S1. Ratio of diameter-to-thickness (D/h) across generations for healthy and PAH baseline models.**

# Womersley Theory for Deformable Walls

Womersley's theory, initially developed for rigid tubes, is extended to address blood flow in deformable vessels where the elasticity of the vessel wall is significant. This theory is particularly useful for capturing wave propagation and phase lag effects in the cardiovascular system under pulsatile flow conditions, providing an analytical framework for understanding the interaction between blood flow and vessel wall motion (Alberto Figueroa et al. 2009; Filonova et al. 2020).

**B.1. Governing Equations and Assumptions**

The following assumptions simplify the analysis:

1. **Axisymmetric Flow**: The flow and wall deformation are considered axisymmetric, meaning there is no dependency on the circumferential direction.
2. **Thin-Walled Elastic Vessel**: The vessel wall is assumed to be thin compared to its radius and behaves as an elastic material, allowing it to deform radially in response to pressure changes.
3. **Small Deformations**: The radial displacement of the wall is small, ensuring linearity in the elastic response.
4. **Oscillatory Flow**: The model assumes a periodic pressure gradient that drives pulsatile blood flow, resulting in oscillatory velocity and pressure fields.

Under these assumptions, we consider the Navier-Stokes equations for incompressible flow in cylindrical coordinates and couple them with equations describing the elastic deformation of the vessel wall.

**B.2. Key Parameters**

1. **Womersley Number α**:

$$\alpha=R\sqrt{\frac{\omega}{\upsilon}}$$

where, R is the vessel radius, ω=2πf is the angular frequency of the oscillatory pressure wave (with f being the frequency), ν is the kinematic viscosity of blood.

The Womersley number characterizes the ratio of inertial to viscous forces. A high α\alphaα indicates that inertial effects dominate, leading to a parabolic velocity profile that flattens in the center of the vessel.

1. **Radial Displacement ξ(r,t):** The vessel wall undergoes radial displacement due to the pulsatile pressure. This displacement is governed by the wall's material properties, specifically the Young's modulus E, wall thickness h, and vessel radius R.

**B.3. Formulation of the Problem**

1. **Navier-Stokes Equations** for Pulsatile Flow: The axial velocity w(r,t) of blood in a cylindrical vessel is described by:

$$\frac{\partial w}{\partial t}= -\frac{1}{\rho}\frac{\partial p}{\partial z} + \upsilon\left( \frac{\partial^{2}w}{\partial r^{2}}+\frac{1}{r}\frac{\partial w}{\partial r} \right)$$

where, p(z,t) is the pressure, ρ is the density of blood, ν is the kinematic viscosity.

1. **Pressure Gradient**: The pressure p(z,t) is assumed to have an oscillatory component, typically given by:

$$p\left( z,t \right)=P_{0}e^{iwt}$$

where $P_{0}$ is the amplitude of the pressure wave, and ω is the angular frequency.

1. **Wall Deformation (Radial Displacement)**: The radial displacement ξ(r,t) of the wall is proportional to the oscillatory pressure applied on the inner wall. The relationship between radial displacement and wall pressure can be derived from the linear elasticity theory as:

$$\xi\left( t \right)=\frac{p\left( z,t \right)R}{Eh}$$

where, E is the Young’s modulus of the vessel wall, h is the wall thickness, R is the vessel radius.

1. **Velocity Profile**: The solution to the Navier-Stokes equation under these assumptions yields a velocity profile in the axial direction, which varies radially and temporally. The complex form of the velocity profile w(r,t) is:

$$w\left( r,t \right)\mathfrak{= R}\left\{ \frac{\rho iw}{P_{0}}\left[ 1-\frac{J_{0}\left( \alpha i \right)}{J_{0}\left( \frac{\alpha ir}{R} \right)} \right]e^{iwt} \right\}$$

where $J_{0}$​ is the zeroth-order Bessel function of the first kind, capturing the radial dependence of the velocity profile.

1. **Circumferential and Axial Stresses**: In the elastic wall, the circumferential $\sigma_{\theta}$ ​ and axial stresses $\sigma_{z}$ are influenced by the pressure-induced deformation:

$$\sigma_{\theta}=\frac{pR}{2h}$$

$$\sigma_{z}=\frac{pR}{4h}$$

where $\sigma_{\theta}$ reflects the hoop stress, and $\sigma_{z}$ represents the longitudinal stress in the vessel wall.

**B.4. Analytical Solution for Pulsatile Flow**

The analytical solution provides insight into the phase difference between the pressure wave and the resulting velocity profile due to inertial effects and vessel compliance. For high Womersley numbers, the centerline velocity lags behind the pressure gradient due to the dominance of inertial forces, while for low Womersley numbers, the velocity profile follows a parabolic shape with minimal phase lag.

The total flow rate Q(t) through the vessel can be obtained by integrating the velocity profile across the cross-section:

$$Q\left( t \right)= \int_{0}^{R} w\left( r,t \right)2\pi rdr$$

This flow rate is phase-shifted relative to the driving pressure wave, a key feature in understanding blood flow dynamics in compliant vessels.

**B.5. Applications and Importance**

Womersley’s deformable wall model has significant applications in cardiovascular modeling, particularly for assessing arterial compliance and wave propagation in large arteries. It is frequently used as a benchmark for verifying computational fluid-structure interaction (FSI) models, where the accuracy of blood flow simulation depends on accurately capturing vessel wall deformation under pulsatile loading.

The theoretical framework provided by Womersley’s model helps to characterize key hemodynamic parameters like wave speed, flow pulsatility, and vascular impedance, which are crucial for understanding conditions like arterial stiffness and hypertension.

# Constrained Mixture Model in a Single Vessel

A single segment of the arterial tree is considered as a thin-walled cylindrical tube composed of three main load-bearing constituents: elastin (*el*), collagen (*col*), and smooth muscle cells (SMCs; *smc*). First, we only consider the passive response of constituents. Each constituent is assumed to separately contribute to the strain energy density:

$$w=w^{el}\left( F_{n}^{el} \right)+w^{smc}\left( F_{n}^{smc} \right)+w^{col}(F_{n}^{col})$$

where $F_{n}^{i}$ is the deformation gradient of each constituent $i\in\{el,smc,col\}$ corresponding to its map from a stress-free configuration to the overall slow-time configuration. We define this deformation gradient as $F_{n}^{i}=F^{s}G^{i}$, where $G^{i}$ is a pre-stretch for each constituent mapping each constituent from its distinct stress-free configuration to the intermediate configuration (Baek et al. 2005). In particular, the pre-stretch mapping for elastin can be expressed as

$$G^{e}=\left[\begin{matrix} G_{r}^{el} & 0 & 0 \\ 0 & G_{\theta}^{el} & 0 \\ 0 & 0 & G_{z}^{el} \end{matrix}\right]$$

where $G_{r}^{el}$, $G_{\theta}^{el}$, and $G_{z}^{el}$ are pre-stretches associated with circumferential and axial directions, and $G_{r}^{el}=1/G_{\theta}^{el}G_{z}^{el}$. Similarly, for collagen fibers and SMCs, $\boldsymbol{M}^{\boldsymbol{i}}$, is defined as the unit vector in the direction of the collagen fiber (*k*) or SMCs. The pre-stretch mappings for collagen and smooth muscle cells are given as

$$G^{k}=G_{h}^{col}M^{k}\otimes M^{k}, G^{smc}=G_{h}^{smc}M^{smc}\otimes M^{smc},$$

where the pre-stretches $G_{h}^{col}$ and $G_{h}^{smc}$ are also called homeostatic stretches, the stretches of the constituents when they are produced. We should note that in the previous applications of growth and remodeling, the pre-stretches were assumed to be constant for a single vessel. In our generalization of the framework to a vascular tree, we account for the variation of pre-stretches across the generations of vessels. Nevertheless, the pre-stretch implies that the homeostatic state in an individual vessel is associated with a constant homeostatic stress for the constituents of the vessel wall.

The orientation of collagen fibers and smooth muscles with respect to the axial direction in their reference configuration, defined by angle $\gamma^{k}$, can be written as

$$\mathbf{M}^{k}=\cos\gamma^{k}\mathbf{e}_{Z}+\sin\gamma^{k}\mathbf{e}_{\theta}, \mathbf{M}^{smc}=\mathbf{e}_{\theta}.$$

For modeling the extension and inflation of a thin wall model, $F^{s}$is considered as $F^{s}=diag[\lambda_{r},\lambda_{\theta},\lambda_{z}]$. The stretch in each constituent $\lambda^{i}$ is expressed in terms of the prestretches using $\mathbf{F}^{i}=\mathbf{F}^{s}\mathbf{G}^{i}$:

$$\lambda_{\theta}^{el}=G_{\theta}^{el}\lambda_{\theta}, \lambda_{z}^{el}=G_{z}^{el}\lambda_{z} \lambda^{k}=G_{h}^{col}\sqrt{\lambda_{\theta}^{2}\sin^{2}\gamma^{k}+\lambda_{z}^{2}\cos^{2}\gamma^{k}}, \lambda^{smc}=G_{h}^{smc}\lambda_{\theta}.$$

The incompressibility of the wall material is imposed by assuming an isochoric motion (i.e.,$\det F^{s}=1$), and thus $\lambda_{r}=\left(\lambda_{\theta}\lambda_{z}\right)^{-1}$. Using the membrane theory (Humphrey 2002), the membrane Cauchy stress (force per deformed length) can be written as

$$\mathbf{T}=\frac{1}{J_{2D}}\mathbf{F}^{s}\frac{\partial w}{\partial\mathbf{F}^{sT}}, T_{\theta\theta}=\frac{1}{\lambda_{z}}\frac{\partial w}{\partial\lambda_{\theta}}, T_{zz}=\frac{1}{\lambda_{\theta}}\frac{\partial w}{\partial\lambda_{z}},$$

where $J_{2D}=\lambda_{\theta}\lambda_{z}.$ The total strain energy per unit area can be written as

$$w=M_{R}^{total}(\nu^{el}\Psi^{el}+\sum_{k} \nu^{k}\Psi^{k}+\nu^{smc}\Psi^{smc}),$$

where $\nu^{el}$, $\nu^{k}$, and $\nu^{smc}$are mass fractions of elastin, collagen fiber families, and SMCs, respectively. In this work, four families of collagen fibers in circumferential, axial, and two diagonal directions were considered with mass fractions $\nu^{k}=(0.1,0.1,0.4,0.4)\nu^{col}$ where $\nu^{col}$ is the total collagen mass fraction(Zeinali-Davarani et al. 2011). The total mass per unit area $M_{R}^{total}$ is the mass of load bearing constituents and can be computed via

where $\rho_{wall}$ is the density of the vascular wall, is the volume fraction of interstitial fluid, and $H$ is the wall thickness under homeostatic conditions.

A neo-Hookean model is employed for the passive elastin response and a Holzapfel exponential model is used for collagen fiber families and passive behavior of circumferentially oriented SMCs

$$\begin{matrix} \Psi^{el}=\frac{c_{1}}{2}\left((\lambda_{\theta}^{el})^{2}+(\lambda_{z}^{el})^{2}+\frac{1}{(\lambda_{\theta}^{el})^{2}(\lambda_{z}^{el})^{2}}-3\right), \\ \Psi^{k}=\frac{c_{2}}{4c_{3}}\left(e^{c_{3}\left((\lambda^{k})^{2}-1\right)^{2}}-1\right), \\ \Psi^{smc}=\frac{c_{4}}{4c_{5}}\left(e^{c_{5}\left((\lambda^{smc})^{2}-1\right)^{2}}-1\right)+\Psi_{act}^{smc}, \end{matrix}$$

where $c_{1}$ is the elastin material parameter, $c_{2}$ and$c_{3}$ are collagen material parameters, and $c_{4}$ and $c_{5}$ are passive SMC material parameters. To include the active tension of vascular SMCs, we use a potential function as given by (Baek et al. 2007)

$$\Psi_{act}^{smc}=\frac{S}{\rho_{wall}}\left(\lambda_{\theta}+\frac{1}{3}\frac{(\lambda_{M}-\tilde{\lambda})^{3}}{(\lambda_{M}-\lambda_{0})^{2}}\right)$$

where $\lambda_{M}$and$\lambda_{0}$ are stretches at which the active force generation is maximum and zero, respectively, and $S$ is the basal active tone. In addition, $\tilde{\lambda}$ is an active stretch of the SMCs in the circumferential direction, which can evolve by SMC remodeling over slow timescales (hours to days). In the current study, we assume$\tilde{\lambda}=\lambda^{smc}$ $.$ Finally, for pressurized thin-walled cylinder with mid-vessel pressure $\bar{p}^{s}$, the force equilibrium in the circumferential direction gives the Laplace law

$$\left.T_{\theta\theta}\right|_{s}=\bar{p}^{s}R\text{ ,}$$

where $R$ is the vessel radius. In our homeostatic optimization framework, we assume the intermediate configuration of the blood vessel in our continuum mechanics formulation is its homeostatic configuration. Therefore, by setting $F^{s}=I$ (i.e., $\lambda_{\theta}=\lambda_{z}=1$) in equations above*,* the homeostatic membrane stress $\mathbf{T}^{i}$ and total stress, $\sigma^{i}=\frac{1}{h^{i}}\mathbf{T}^{i},$ can be determined as a function the passive material properties ($c_{1}-c{}_{5}$), constituent prestretches ($G_{r}^{el}$, $G_{\theta}^{el}$,$G_{h}^{col}$, and$G_{h}^{smc}$) and their mass fractions $\nu^{i}$, and active SMC parameters, $\lambda_{M}$, $\lambda_{0}$and$S$.

# Slow-time hemodynamics

Here we present an algorithm that computes the slow-time pressure and flow at each vessel for a given bifurcating tree. We introduce the index $[k,m]$ to label an individual vessel within the tree generation $k$ for $m=1,..2^{k-1}$. A large sparse matrix can be constructed to account for the pressure continuity and flow conservation at each bifurcation as well as Poiseuille flow resistance at each vessel. The block-matrix, related to the vessel $[k,m]$, comprises of flow splits and Poiseuille equation for daughter vessels in generation $k+1$, as shown below

$$\begin{matrix} Given resistance\text{ }res[k,m]: \\ Construct [k,m] -block of equations: \\ \text{ }q^{s}[k+1,2m-1]+q^{s}[k+1,2m]=q^{s}[k,\mathrm{m}] \\ \text{ }p_{T}^{s}[k,\mathrm{m}]-p_{T}^{s}[k+1,2m-1]=res[k+1,2m-1]q^{s}[k+1,2m-1] \\ \text{ }p_{T}^{s}[k,\mathrm{m}]-p_{T}^{s}[k+1,2m]=res[k+1,2m]q^{s}[k+1,2m] \\ for uknowns: \\ q^{s}[k,\mathrm{m}],q^{s}[k+1,2m-1],q^{s}[k+1,2m],p_{T}^{s}[k,\mathrm{m}],p_{T}^{s}[k+1,2m-1],p_{T}^{s}[k+1,2m] \end{matrix}$$

For boundary conditions in the slow-time hemodynamic system in a tree, we consider two options: (1) given input steady flow at the root vessel and terminal steady pressure at outlets; and (2) given input steady pressure at root vessel and steady flow at outlets. In the manuscript, for the numerical example of the symmetric tree we consider boundary conditions (1) while for the asymmetric tree example (2).

# Fast-time Hemodynamics

Here we present an algorithm that for a given geometry of a bifurcating tree and discrete frequency $\omega_{n}$ computes the impedance and then fast-time hemodynamics at each vessel. First, we use the following relations between characteristic $Z^{c}$, terminal $Z^{T}$, and input $Z^{inp}$ impedance of each vessel, and the reflection coefficient $\Gamma$

$$\begin{matrix} \Gamma=\frac{Z^{T}-Z^{c}}{Z^{T}+Z^{c}}, \\ Z_{p}^{T}=\left(\frac{1}{Z_{d1}^{inp}}+\frac{1}{Z_{d2}^{inp}}\right)^{-1}, (\text{p: parent} \text{vessel,} \text{d1} \text{and} \text{d2:} \text{daughter} \text{vessels),} \\ Z^{inp}=Z^{c}\frac{1+\Gamma e^{-i\omega2L/c}}{1-\Gamma e^{-i\omega2L/c}}, \end{matrix}$$

where$L$ and $c$ are vessel length and wave velocity, respectively. First, we compute the impedance at each vessel from bottom-to-top of the tree. We consider that the terminal impedance or, equivalently, the reflection coefficients $\Gamma$ at outlets are given

Second, we reconstruct the pulsatile hemodynamics in the system by computing the pressure and flow solution at each vessel from top-to-bottom of the tree. The components of the fast-time terminal pressure $P_{n}^{T}$ and input flow $Q_{n}^{inp}$ in frequency domain are found using from Womersley’s solution. For illustration of the algorithm, we consider the boundary condition at the root vessel as a given input pressure.

$$\begin{matrix} Given:P_{n}^{inp}[1,1] \\ \text{ }R,L,\omega_{n},c_{n},\Gamma_{n},Z_{n}^{c},Z_{n}^{inp} at vessel [k,m] \\ Find:P_{n}^{T}[k,m],Q_{n}^{inp}[k,m]\text{ } \\ \text{for }k=1:N \\ \text{ for }m=1:2^{k-1} \\ \text{ }\bar{m}=\text{round}(m/2) \\ \text{ }P_{n}^{inp}[k,m]=P_{n}^{T}[k-1,\bar{m}]\text{ (}for k>1\text{)} \\ \text{ }Q_{n}^{inp}[k,m]=P_{n}^{inp}[k,m]/Z_{n}^{inp}[k,m] \\ \text{ }H_{forw}=P_{n}^{inp}[k,m]/\left(1+\Gamma_{n}[k,m]e^{-i2\omega_{n}L[k,m]/c_{n}[k,m]}\right) \\ \text{ }P_{n}^{T}[k,m]=H_{forw}e^{-i\omega_{n}L/c_{n}[k,m]}\left(1+\Gamma_{n}[k,m]\right) \\ \text{ end} \\ \text{end} \end{matrix}$$

# References

Alberto Figueroa C, Baek S, Taylor CA, Humphrey JD (2009) A computational framework for fluid–solid-growth modeling in cardiovascular simulations. Comput Methods Appl Mech Eng 198:3583–3602. https://doi.org/10.1016/J.CMA.2008.09.013

Baek S, Rajagopal KR, Humphrey JD (2005) A Theoretical Model of Enlarging Intracranial Fusiform Aneurysms. J Biomech Eng 128:142–149. https://doi.org/10.1115/1.2132374

Baek S, Valentín A, Humphrey JD (2007) Biochemomechanics of Cerebral Vasospasm and its Resolution: II. Constitutive Relations and Model Simulations | SpringerLink. Ann Biomed Eng 35:1498. https://doi.org/10.1007/s10439-007-9322-x

Filonova V, Arthurs CJ, Vignon-Clementel IE, Figueroa CA (2020) Verification of the coupled-momentum method with Womersley’s Deformable Wall analytical solution. Int J Numer Method Biomed Eng 36:e3266. https://doi.org/https://doi.org/10.1002/cnm.3266

Gharahi H, Filonova V, Mullagura HN, et al (2023) A multiscale framework for defining homeostasis in distal vascular trees: applications to the pulmonary circulation. Biomech Model Mechanobiol 22:971–986. https://doi.org/10.1007/s10237-023-01693-7

Humphrey JD (2002) Cardiovascular Solid Mechanics: Cells, Tissues, and Organs. Springer-Verlag, New York

Zeinali-Davarani S, Raguin LG, Vorp DA, Baek S (2011) Identification of in vivo material and geometric parameters of a human aorta: toward patient-specific modeling of abdominal aortic aneurysm. Biomech Model Mechanobiol 10:689–699. https://doi.org/10.1007/s10237-010-0266-y
